# Design and Experimental Validation of a Photocatalyst Recommender Based on a Large Language Model

**DOI:** 10.1002/anie.202514544

**Published:** 2025-12-09

**Authors:** Francis Millward, Michał Kulczykowski, Jay Badland‐Shaw, Sara Szymkuc, Rajan Suraksha, Aniket Kumar Srivastawa, Violaine Manet, Máire Griffin, Megan Bryden, Thomas Comerford, Lea Hämmerling, Aminata Mariko, Bartosz A. Grzybowski, Eli Zysman‐Colman

**Affiliations:** ^1^ Organic Semiconductor Centre, EaStCHEM School of Chemistry University of St Andrews St Andrews KY16 9ST UK; ^2^ Allchemy, Inc. Highland Indiana 46322 USA; ^3^ Institute of Organic Chemistry Polish Academy of Science ul. Kasprzaka 44/52 Warsaw 02–224 Poland; ^4^ Center for Algorithmic and Robotized Synthesis (CARS) of Korea's Institute for Basic Science (IBS) and Department of Chemistry Ulsan National Institute of Science and Technology 50 UNIST‐gil, Eonyang‐eup, Ulju‐gun Ulsan South Korea

**Keywords:** Large language models, Machine learning, Photocatalysis

## Abstract

Utilizing an extensive library of literature on photocatalytic transformations, we disclose the development of a machine learning (ML) model for the recommendation of photocatalysts most suitable for reactions of interest. The model is trained on > 36 000 such literature examples and uses an architecture inspired by the Bidirectional Encoder Representations from Transformer (BERT) large language model. Under cross‐validation, it can suggest the “correct” photocatalysts with ∼90% accuracy. When experimentally tested on five out‐of‐box reactions, this algorithm consistently suggested photocatalysts that gave yields competitive to those chosen by human researchers and frequently suggested alternative photocatalysts that are potentially more appealing than the originally selected photocatalyst. Altogether, this platform serves as a valuable tool for researchers undertaking reaction optimization programs. The model is free to use at https://photocatals.grzybowskigroup.pl/predict/.

## Graphical abstract

Choosing a photocatalyst for a given reaction can be challenging due to complex mechanisms and multiple parameters that govern the outcome of a photocatalyzed reaction. Herein, we disclose a machine learning (ML) model that can suggest catalysts for a given reaction using an online portal. The model was experimentally validated against five photocatalysis reactions, in all cases suggesting productive photocatalysts. This model serves as a valuable tool for researchers optimizing photocatalysis reactions.

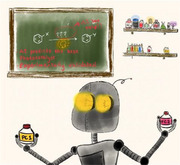

## Introduction

Machine Learning (ML) algorithms are at the heart of the ongoing AI revolution. They are impacting numerous areas of chemical research, from protein design^[^ ^1^ ^]^ to synthetic chemistry^[^ ^2^, ^3^, ^4^, ^5^, ^6^, ^7^ ^]^ to catalysis. In the latter context, there have been notable recent studies using ML to design homogeneous catalysts offering improved performance. For instance, Sigman and coworkers pioneered data‐driven workflows and regression models to design catalysts offering improved enantiomeric outcomes^[^ ^8^, ^9^, ^10^ ^]^ and, in some *tour de force* demonstrations, industrially relevant scalability.^[^ ^11^ ^]^ ML models to find new ligands have been developed by the Denmark,^[^ ^12^, ^13^ ^]^ Ackermann,^[^ ^14^ ^]^ and List and Varnek^[^ ^15^ ^]^ groups and have been based on both 2D and 3D featurization. Schoenebeck and coworkers extended these efforts to dinuclear catalysts,^[^ ^16^ ^]^ whereas Hong and coworkers demonstrated systems that seek metal replacements.^[^ ^17^ ^]^

In parallel to these studies seeking unprecedented catalysts, there is also a desire for ML recommender systems that would suggest the most suitable *known* catalyst for a particular transformation. In a recent study from one of our groups, we described a proof‐of‐principle model of this kind that used a standard multilayer perceptron (MLP) architecture to propose Mg‐based catalysts with an accuracy of around 80%.^[^ ^18^ ^]^ This study was accompanied by experiments that validated most—but not all—predictions.

Herein, we disclose the development and experimental validation of an efficient recommender of photocatalysts for visible light‐driven organic synthesis reactions. Photocatalysis is now a burgeoning area of research, with rapidly expanding applications in numerous sectors, such as the pharmaceutical industry.^[^ ^19^, ^20^, ^21^ ^]^ ML has seen some uptake in the photocatalysis community; e.g., Glorius et al. developed an ML platform for guiding substrate discovery in energy transfer catalysis,^[^ ^22^ ^]^ while Noto, Saito, and coauthors have reported the prediction of organic photocatalyst performance in both [2+2] cycloadditions and the nickel co‐catalyzed metallaphotoredox synthesis of phenols.^[^ ^23^, ^24^ ^]^

Still, when it comes to selecting the most suitable photocatalyst for a given reaction during optimization campaigns, thermodynamic parameters such as redox potentials and excited‐state energies are often not predictive of reaction outcomes, as photocatalysts tend to degrade,^[^ ^25^ ^]^ and extensive photocatalyst screening programs are necessary. Furthermore, the motivations for the choice of specific photocatalysts surveyed are rarely discussed, prompting questions about whether the identified conditions are premeditated or opportunistic (according to photocatalyst availability and/or popularity/bias in prior literature^[^ ^26^ ^]^), and whether better photocatalysts for a given reaction of interest can be found. To this end, we disclose here the development of a photocatalyst recommender that suggests photocatalysts likely to catalyze a given reaction.

We first tested an ML recommender akin to our previously disclosed platform^[^ ^18^ ^]^—similar to the said reference, it achieves an accuracy of around 80%. We then sought to improve this metric using a transformer architecture previously used in large language models. By first pre‐training this transformer on a large collection of reactions (not necessarily photocatalytic), we taught it to understand the general syntax of molecular representations, which, in turn, proved beneficial in training the ultimate model for photocatalyst prediction. This architecture offers significantly improved accuracy (∼90%). This model was then used to suggest alternative photocatalysts for several literature reactions that were described in the literature only after the development of our model and were therefore not in its training/test set. The results of these studies are encouraging in that all photocatalysts suggested by the recommender gave appreciable yields, and in four out of five cases, the model identified at least one photocatalyst that performs competitively with the “optimal” one identified in the original publication or with photocatalysts chosen by a human researcher.

## Results and Discussion

### Data Curation

We began by collecting literature data for model training. Having identified 31 archetypal photocatalysts (see Supplementary Information and subset in Figure 2), we queried the Reaxys database for reactions using these photocatalysts. Following filtering of the data (see Supporting Information for details), a dataset of 36097 unique reactions remained. It should be noted that the data set is heavily imbalanced, reflecting the prevailing historical bias in the choice of photocatalysts, where some were associated with >7000 reactions, while others with a mere 20. The photocatalysts were one‐hot encoded (that is, given unique labels), whereas the reactions were represented in two ways: i) by the concatenated SMILES of the reaction substrates and ii) by the reaction cores (that is, atoms that change their bonding patterns and flanking atoms to within a bond‐distance radius of 3) that were extracted after atom‐mapping the reactions using our MAPPET algorithm.^[^ ^27^, ^28^ ^]^ Altogether, 4854 distinctive reaction cores/types were identified. It should be noted that the model did not incorporate information about the presence or choice of co‐catalyst, base, solvent, reaction time, excitation wavelength, photon flux, and other reaction parameters. In Ref. [18], we showed that automatic extraction of the relevant information from Reaxys is problematic due to errors and differences in nomenclature. In the said reference, we were able to curate some of this data manually by consulting ∼1500 source publications—in the present case, however, such manual curation for >36 000 entries would be prohibitive. In a broader context, we ^[^ ^26^, ^29^, ^30^ ^]^ and others^[^ ^31^, ^32^, ^33^, ^34^ ^]^ have previously shown that prediction of reaction conditions and/or yields is extremely difficult due to the historical biases in the data and, for yields, scarcity of negative examples.

With these preliminaries and limitations, the overall learning strategy was to use both the substrate and reaction‐core SMILES. As we previously showed,^[^ ^18^ ^]^ this approach is not redundant, as both the reaction type and the specifics of the reaction (e.g., functional groups present in the substrates outside of the core) independently contribute to the prediction outcome. We tested two types of models, an MLP and a Bidirectional Encoder Representations from Transformer (BERT)‐based language model.

### The MLP Model

**Figure 1:**
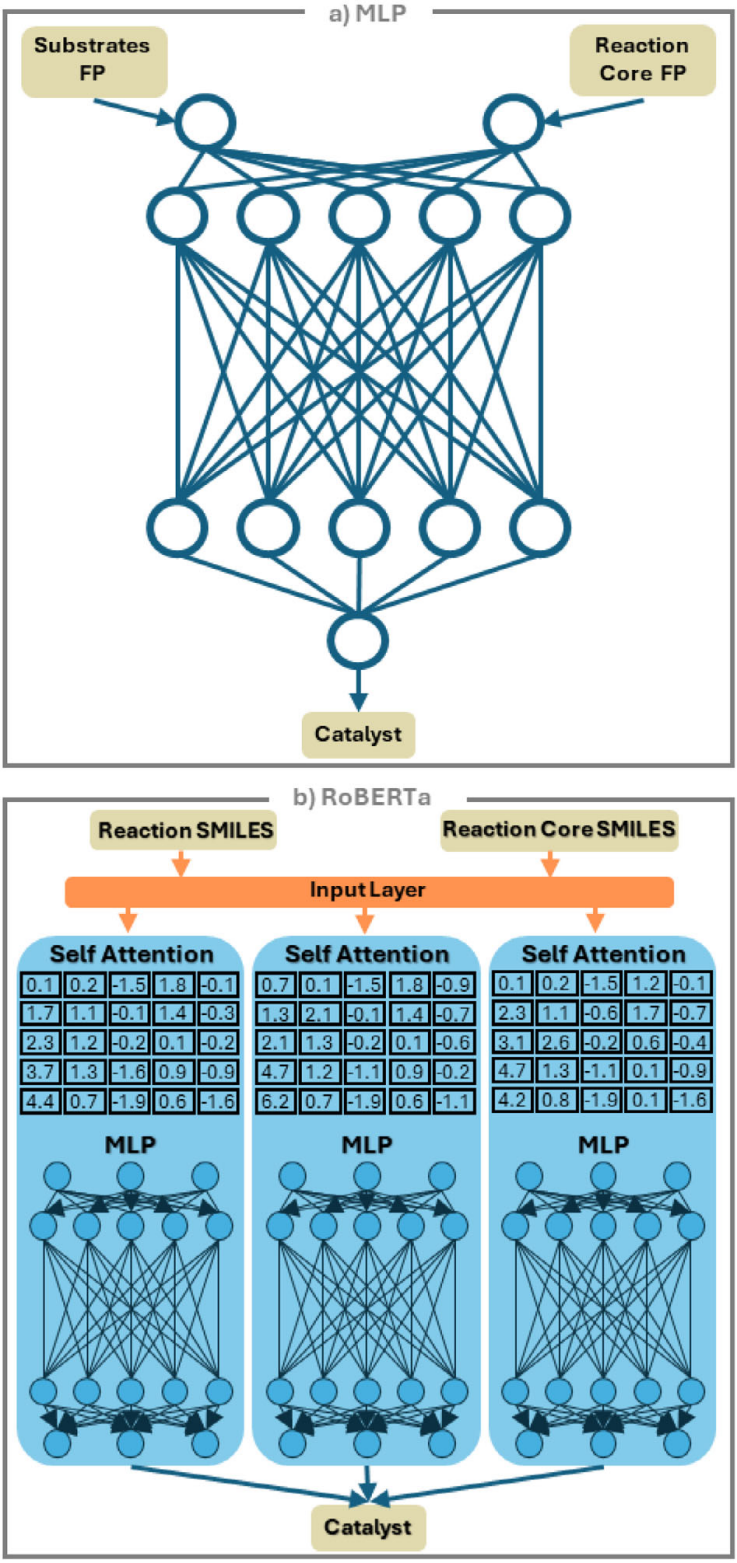
The architectures of a) the multilayered perceptron, MLP, model and b) the language model based on the RoBERTa extension of BERT. The essence of the BERT (Bidirectional Encoder Representations from Transformer) approach is a self‐attention mechanism, which, in the case of SMILES strings, enables the model to capture complex relationships between molecular substructures across long distances by trying to put attention on how tokens relate to one another. The bidirectional understanding is particularly valuable for chemical reactions, where reactivity often depends on “interactions” between functional groups that may be separated by multiple atoms. Pre‐training on a large, unlabeled dataset helps the model to understand principles of chemistry, which increases performance on supervised fine‐tuning tasks where data is scarce and allows for better generalization to unseen data during inference.

(Figure 1a) used two inputs: the substrates SMILES represented as a standard Morgan fingerprint of size 64 and, for the reaction cores, a Differential Reaction Fingerprint (DRFP)^[^ ^35^ ^]^ with a vector size of 64 and radius 3. Each input was processed through a distinct hidden layer, the outputs of which were subsequently concatenated. This concatenated layer was followed by two additional hidden layers and a final output layer, which yielded a 31‐dimensional vector of logits corresponding to the numbers/labels of one‐hot‐encoded photocatalysts present in the dataset. This vector was transformed into the model's final rankings via the *softmax* function. The model's hyperparameters, including the activation function, dimensions of the hidden layers, kernel initializers, and the method for merging the inputs at the hidden layer, were optimized using the Optuna library.

**Figure 2:**
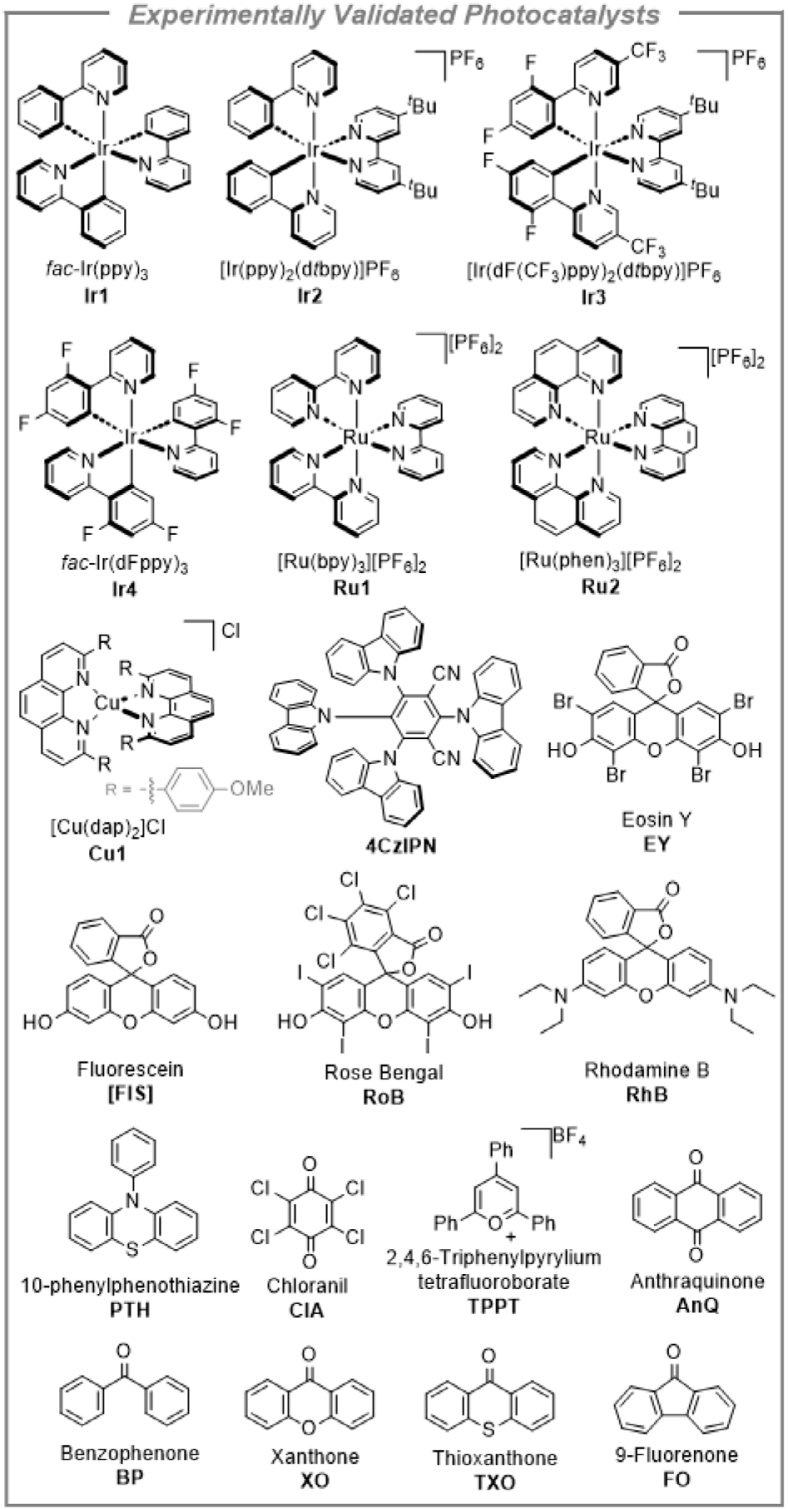
A subset of 31 photocatalysts used to train the ML model (for the full set, please consult the Supporting Information). For any given reaction, the model always ranks all 31 catalysts. Here, for each of the reactions committed to experimental validation, the top‐5 suggestions were taken. Across five reactions tested, 19 catalysts shown here were among the top‐5 choices; the 20^th^ catalyst, **Ir3**, was never suggested among the top‐5 but was used as a control chosen by human researchers.

To address dataset imbalance, the model was trained using a weighted cross‐entropy loss. Model validation was carried out using a 5‐fold cross‐validation and leveraging three performance metrics to ensure appropriate training: accuracy, F1 score with weighted averaging, and F1 score with macro averaging. The accuracy and F1 scores were, respectively, 79.0% ± 1.6% and 79.1% ± 1.6%, while F1 with macro averaging was ∼71.5% ± 1.5%. Performance was further evaluated using the top‐*k* metric (that assesses the model's ability to predict the ground truth photocatalyst within its top *k* predictions). The correct photocatalyst was chosen by the model as its top prediction (top‐1) in 79% of cases, within the top‐4 predictions in ∼95% of cases, and within the top‐10, in 99% of cases.

### BERT‐Based Model

(Figure 1b). The aforementioned performance of the MLP model was on par with a conceptually similar architecture we recently developed to predict one‐hot‐encoded Mg‐based catalysts most suitable for a given reaction.^[^ ^18^ ^]^ However, we sought to improve these metrics and therefore considered another approach specifically designed to learn language representations, here the syntax of SMILES strings. This model uses RoBERTa,^[^ ^36^ ^]^ a variant of Google's BERT^[^ ^37^ ^]^ that is fine‐tuned for optimal hyperparameters. BERT, an advanced ML model for natural language processing tasks, introduces a transformer‐based architecture that leverages bidirectional training. This innovative approach contrasts with traditional unidirectional models by considering contextual influences from both the “left” and the “right” directions within a given text. RoBERTa, an enhancement developed by Facebook AI, refines BERT's performance further. It uses architecture illustrated in Figure 1b and was pre‐trained on 1715395 reactions taken mostly from the Open Reaction Database (https://open‐reaction‐database.org/) and standardized using the MolVS library. Analogously to the photocatalyst dataset, reaction cores were extracted and combined with reaction SMILES, forming a two‐sentence input to the model. The ByteTokenizer, trained on this dataset, tokenized the input SMILES into distinctive classes from the trained tokenizer dictionary. Each token was assigned an integer value corresponding to that token text value in the vocabulary. The input vector dimension was set to 1024 elements, with 15% of all inputs not representing special tokens statically masked. The model was then trained over two epochs to predict these missing parts and reconstruct the ground‐truth SMILES. This pre‐training was important for the model to learn the “grammar” of correct SMILES describing reactions and pinpointing elements of the reactants that undergo reaction, thus gaining “intuition” about the reaction process (see also caption to Figure 1b).

Subsequently, the model underwent fine‐tuning to perform the actual task of interest—that is, to assign each of the reactions from the original 36097 set to one of the 31 photocatalysts. This fine‐tuning phase spanned 10 epochs. Under five‐fold cross validation, this pre‐trained model performed significantly better than MLP: both accuracy and F1 were 90.0% ± 0.4% while F1 with macro averaging was ∼ 84% ± 0.6%. The top‐k statistics also improved, as the ground‐truth catalyst was the top‐1 prediction in 89.99% of cases, within the top‐2 predictions in 95.7% of cases, and within the top‐7 in 99% of cases.

### WebApp Predictor

To facilitate wider use of the model, we developed a web application freely available at https://photocatals.grzybowskigroup.pl/predict/. The WebApp is straightforward to use. The SMILES code for a given reaction is copied into the search box, and the model reduces the scheme to a simplified reaction group SMILES. Only the starting substrates and product, separated by a reaction arrow, are required; considerations of co‐catalysts and other additives are not included in the model's design (see Supporting Information). The top‐5 photocatalysts are then suggested, ranked by the model's probability/confidence rating that the photocatalyst will successfully catalyze the reaction. It is important to note that the confidence rating is an internal metric of the model and, as demonstrated below, does not correlate with experimental yields, though every top‐5 recommendation made by the recommender was effective in catalyzing the reactions discussed in the remaining part of the paper.

### Experimental Validation

We proceeded to validate the model. In doing so, we note that the model will suggest photocatalysts for *any and all* reactions, even those that cannot be performed photocatalytically—this is a well‐known limitation of models in which catalysts are one‐hot‐encoded. Accordingly, we focus on literature‐precedented reactions that were previously shown to be suitable for photocatalysis but were not included in the training‐set data. The model was trained on reactions published up until the end of 2021; therefore, reactions for the experimental screen used publications from 2022 or later. We then assessed whether the ML model would suggest photocatalysts that are i) competitive (that is, equivalent or higher yielding) with the optimal photocatalyst identified in the original publication and ii) better than photocatalysts that were not among the model's top‐five suggestions but were selected by human researchers. The human researcher photocatalyst selections included at least two of the following photocatalysts: **4CzIPN**, [Ir(dF(CF_3_)ppy)_2_dtbbpy](PF_6_) (**Ir3**), *fac*‐Ir(ppy)_3_ (**Ir1**), or [Ru(bpy)_3_](PF_6_)_2_ (**Ru1**). These photocatalysts are widely employed within screening campaigns and are all commercially available, and were thus included in the trials to benchmark the performance of the ML model. This ML model only suggests the ionic form of charged photocatalysts and does not consider the counterion; in situations where these were suggested, the most commonly used counterions for the photocatalysts were employed. SMILES for the validation reactions are provided in the Supporting Information as example inputs for using the WebApp.

### ATRA Reaction

**Figure 3:**
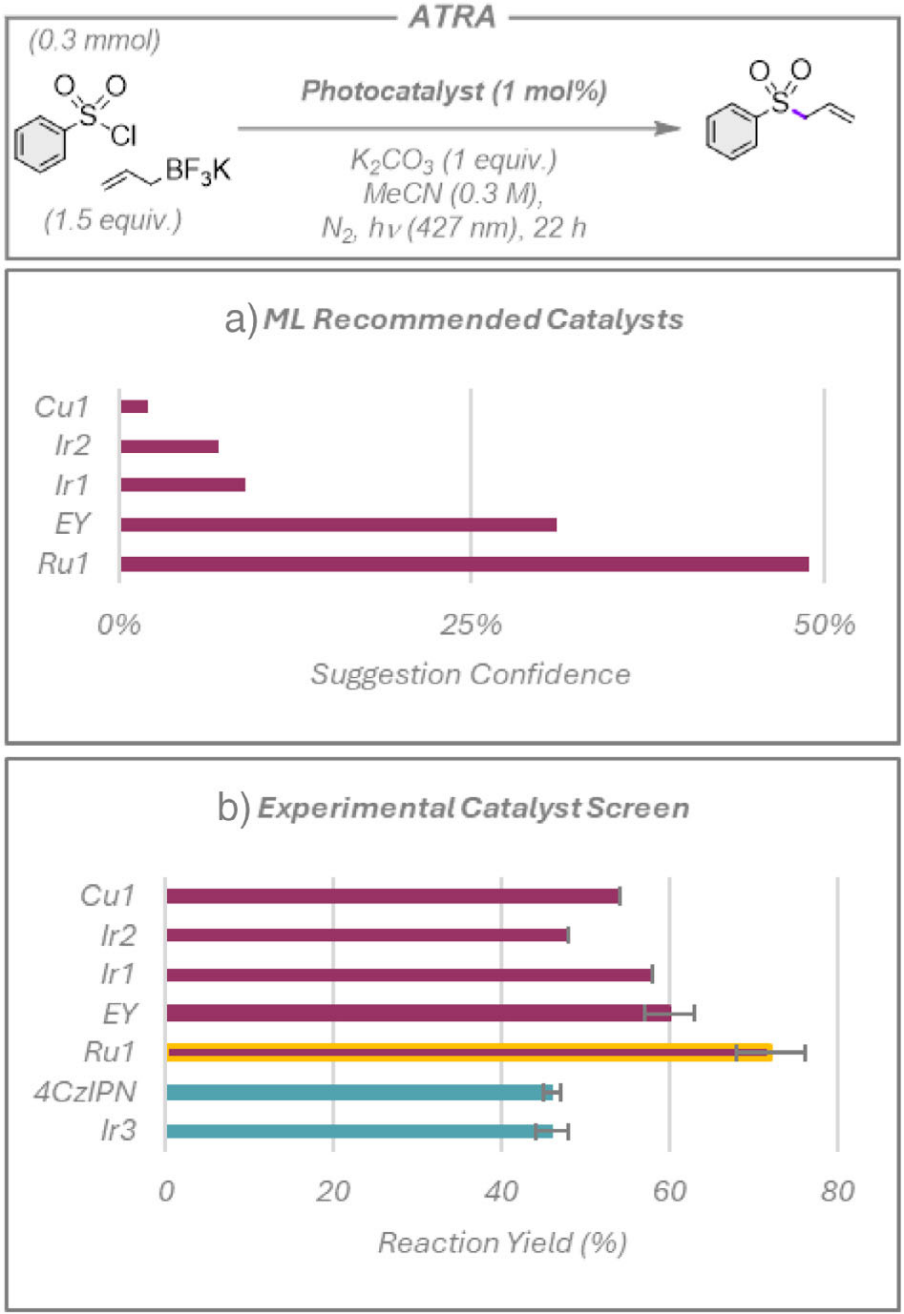
ATRA reaction with phenylsulfonyl chloride. a) Top five photocatalysts suggested by the model and ranked by confidence rating. b) Experimental yields. Red bars show the photocatalysts suggested by the model, with the yellow outline highlighting a previously untested yet productive photocatalyst, **Ru1**, which the ML model identified. Blue bars represent additional photocatalysts chosen by human researchers. Yields presented are the average of two separate quantitative ^1^H NMR experiment repeats, using 1,3,5‐trimethoxybenzene as an internal standard, with the error bars indicating the standard deviation.

We began by exploring the atom transfer radical addition (ATRA) reaction of phenylsulfonyl chloride to potassium allyltrifluoroborate, Figure 3.^[^ ^38^ ^]^ The original paper evaluated **4CzIPN**, **Ir3**, [Ir(ppy)_2_dtbbpy](PF_6_) (**Ir2**), Eosin Y (**EY**), and **Ir1**, identifying the latter as the optimal photocatalyst in their setup.^[^ ^38^ ^]^ Our algorithm ranked **Ir1** among the top five hits for this reaction; however, it was more confident in the use of both **Ru1** and **EY**, while **Ir2** and [Cu(dap)]Cl_2_ (**Cu1**) were also suggested. All five photocatalysts suggested by the model promoted the reaction, affording the desired product in 48%–72% yield (Figure 3). Most notably, the top‐ranked **Ru1**, which was not screened in the original publication, gave the highest yield of the photocatalysts tested. The use of **EY**, a more sustainable organic photocatalyst, ranked by the ML model as the second most likely to work, was also productive using our photoreactors, achieving comparable yields to the more expensive **Ir1**. Interestingly, the authors reported that **EY** did not work as a photocatalyst, which is in contrast to our experiments and highlights the impact of the reaction setup (e.g., light source, photon flux, etc.) in impacting reaction yields. We also assessed **Ir3** and **4CzIPN**, which are human researcher choices, as they are two of the most popularly screened photocatalysts for transformations requiring balanced redox potentials and are frequently included in photocatalyst library screens. Both performed adequately (each affording yields of 46%), though they performed worse than all of the ML‐predicted catalysts. This first reaction clearly showcases the potential utility of the disclosed ML model: the suggested photocatalysts all worked, and the ML model identified **Ru1**, which outperformed all other photocatalysts screened, and was not considered in the photocatalyst optimization program in the initial publication.^[^ ^38^ ^]^

**Figure 4:**
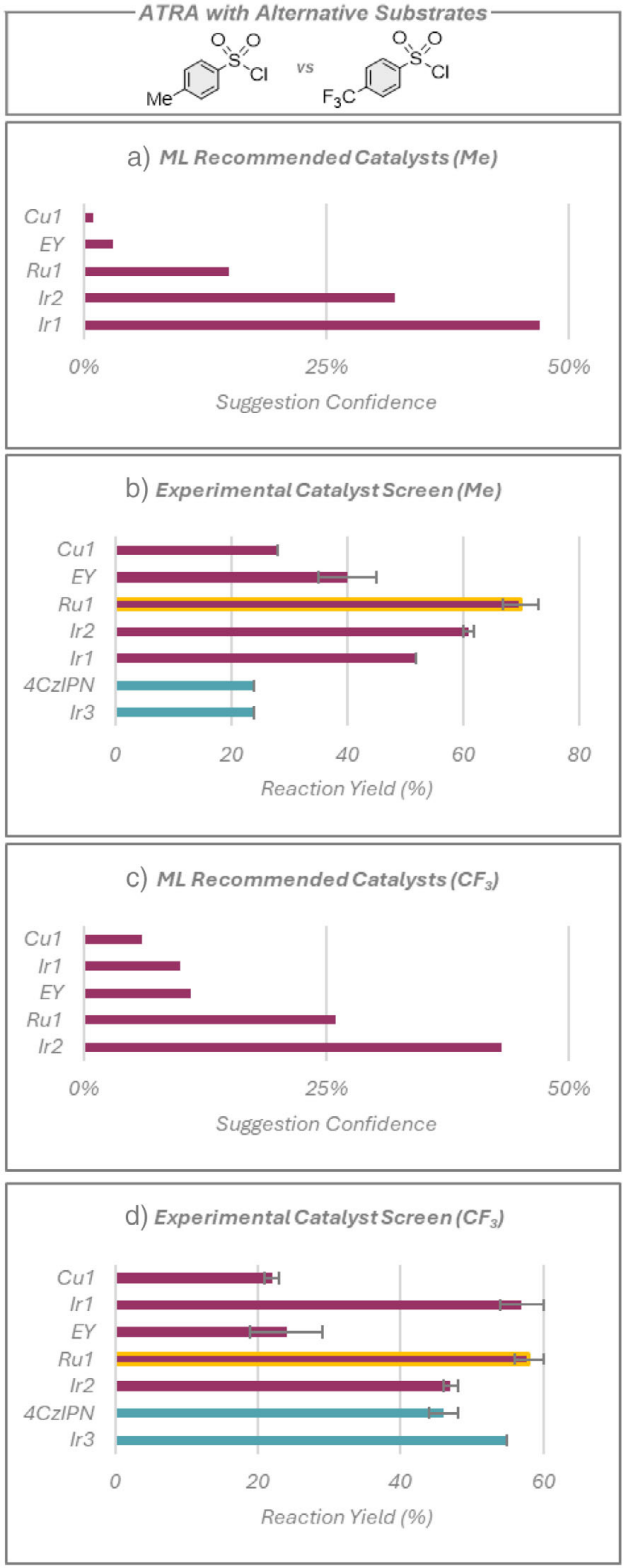
ATRA reaction using alternative substrates. a) Top five RoBERTa‐suggested photocatalysts ranked by confidence rating with *p*‐toluenesulfonyl chloride. b) Experimental yields with *p*‐toluenesulfonyl chloride. a) Top five photocatalysts suggested by the ML model and ranked by confidence rating with *p*‐trifluoromethylphenylsulfonyl chloride. b) Experimental yields with *p*‐trifluoromethylphenylsulfonyl chloride. Red bars show the photocatalysts suggested by the model, with the yellow outline highlighting a previously untested yet highly productive photocatalyst, **Ru1**, which the ML model identified. Blue bars represent additional photocatalysts chosen by human researchers. Yields presented are the average of two separate quantitative ^1^H NMR experiment repeats, using 1,3,5‐trimethoxybenzene as an internal standard, with the error bars indicating the standard deviation.

We next probed how the choice of substrate could influence the ML model's recommendation. The same five photocatalysts were suggested for *p*‐toluenesulfonyl chloride, or *p*‐trifluoromethylphenylsulfonyl chloride, Figure 4; however, there was a significant difference in the order and confidence ratings outputted by the ML for each of these substrates. Again, **Ru1** afforded the highest experimental yields of 70% and 58%, further evidencing the value of utilizing this ML model for photocatalyst selection.

### Phosphorylation

**Figure 5:**
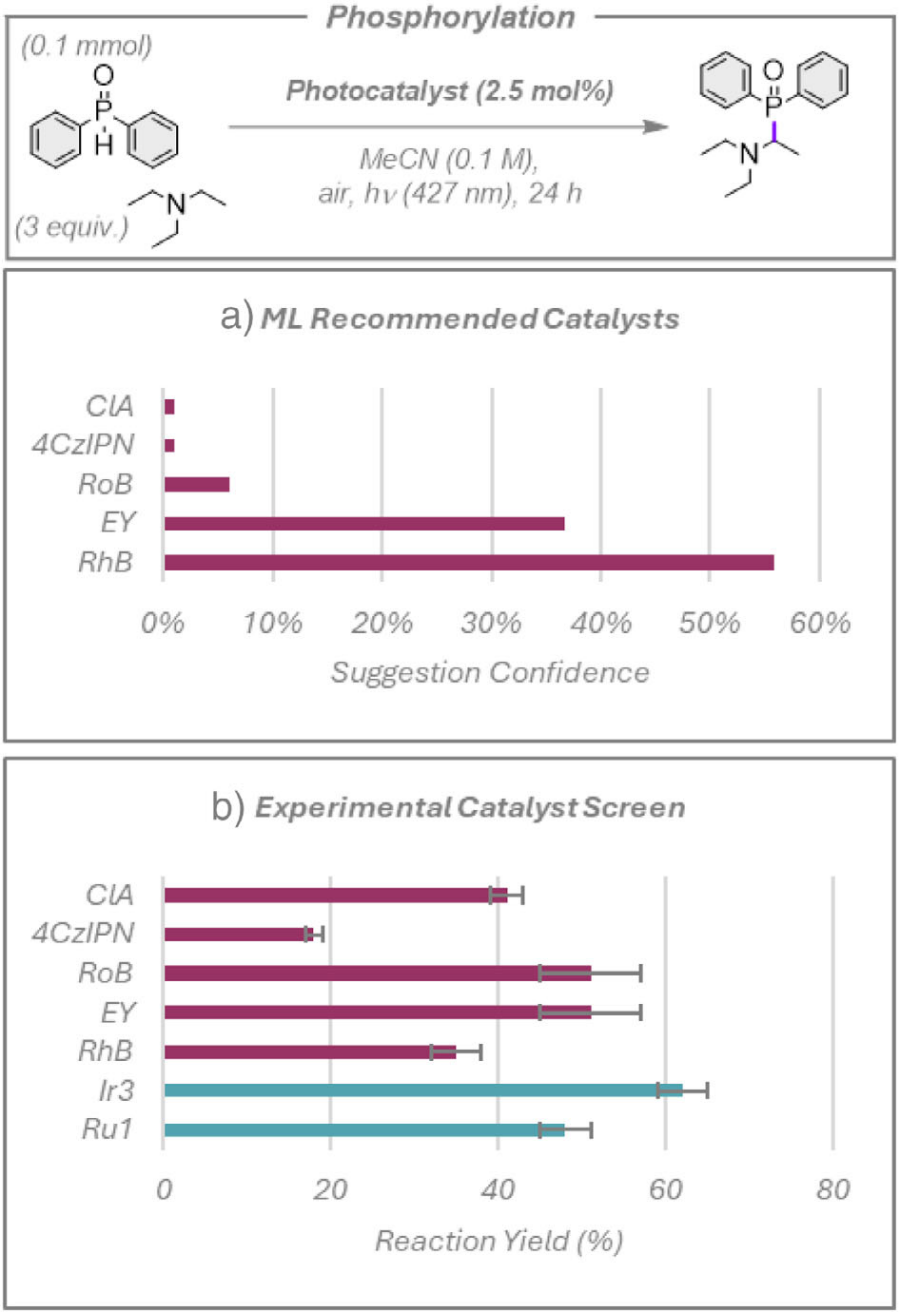
Photocatalyzed phosphorylation of triethylamine. a) Top five photocatalysts suggested by the model and ranked by confidence rating. b) Experimental yields. Red bars show the catalysts suggested by the model. Blue bars represent additional photocatalysts chosen by human researchers. Yields presented are the average of two separate quantitative ^1^H NMR experiment repeats, using 1,3,5‐trimethoxybenzene as an internal standard, with the error bars indicating the standard deviation.

We next explored the photocatalyzed phosphorylation of tertiary amines, using triethylamine as a model substrate, Figure 5.^[^ ^39^ ^]^ The authors used a bespoke photocatalyst, [Ru(CF_3_‐bpy)_2_(OMe‐bpy)](PF_6_)_2_, which was not accessible to us nor included in the library of photocatalysts available to the ML model; thus, this photocatalyst was not tested. The algorithm heavily favored organic photocatalysts in its recommendation for this phosphorylation reaction, providing high confidence ratings to **EY** and Rhodamine B (**RhB**) and lower confidence suggestions to chloranil (**ClA**), **4CzIPN**, and Rose Bengal (**RoB**). All five photocatalysts promoted the reaction in 35%–51% (Figure 5), which is to be expected, as the key photocatalytic step in this reaction is the oxidation of the tertiary amine (*E_ox_*_(triethylamine)_ = 0.83 V versus SCE in MeCN).^[^ ^40^ ^]^ We also assessed **Ir3** and **Ru1** as the human researcher choices, which can both also oxidize triethylamine, with the former giving the highest yield of 62% of the seven photocatalysts tested. While the ML model consistently suggested viable photocatalysts for this reaction, the current version did not identify the photocatalyst that produced the highest yield.

### Dealkylative Acylation

**Figure 6:**
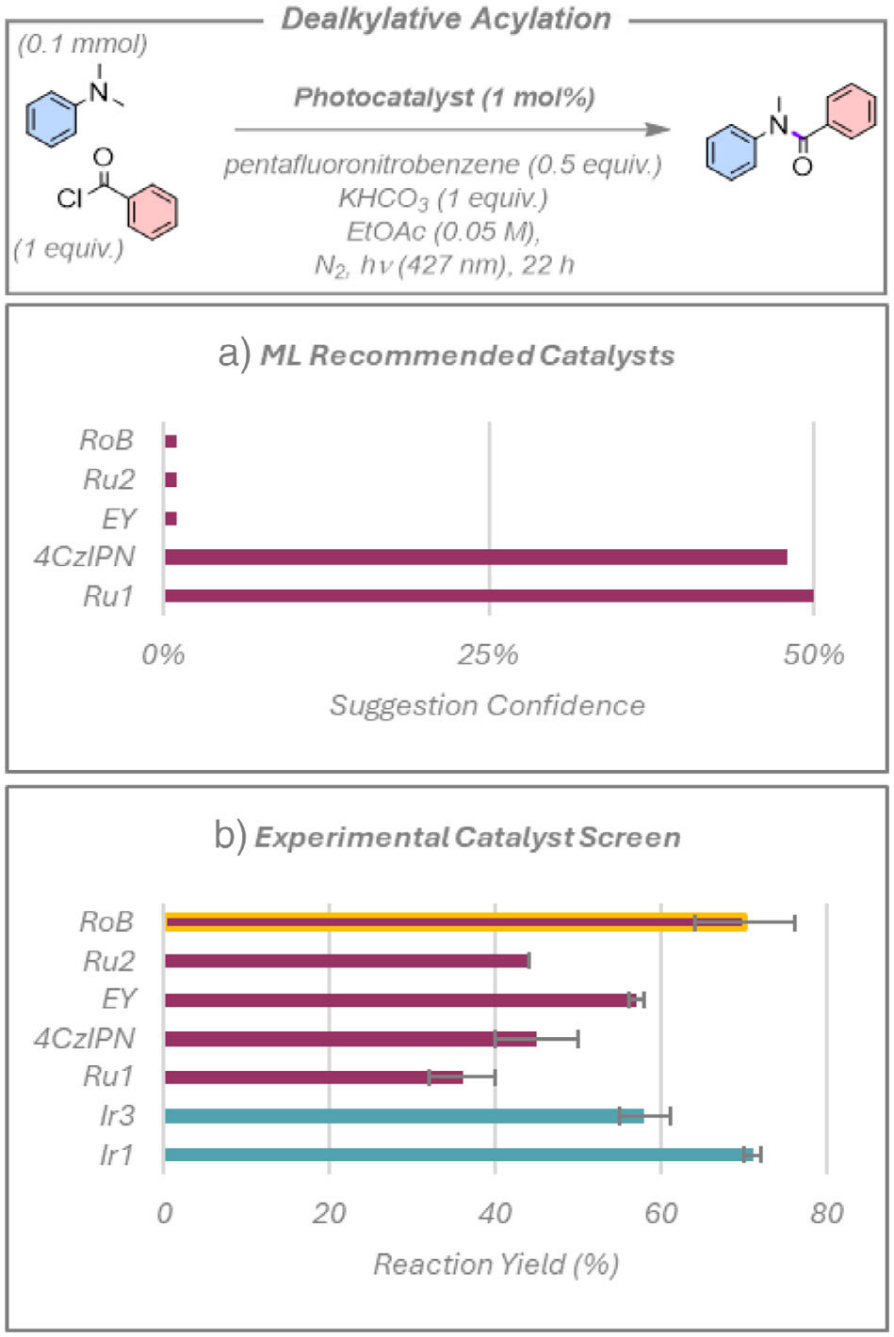
Photocatalyzed dealkylative acylation. a) Top five photocatalysts suggested by the model and ranked by confidence rating. b) Experimental yields. Red bars show the photocatalysts suggested by the model, with the yellow outline highlighting a previously untested yet productive photocatalyst, **RoB**, which the ML model identified. Blue bars represent additional photocatalysts chosen by human researchers. Yields presented are the average of two separate quantitative ^1^H NMR experiment repeats, using 1,4‐bis(trimethylsilyl)benzene as an internal standard, with the error bars indicating the standard deviation.

We next explored the dealkylative amide formation from alkyl amines, Figure 6.^[^ ^41^ ^]^ The proposed reaction mechanism involves the in situ formation of a secondary amine, driven by pentafluoronitrobenzene acting as a hydrogen atom transfer (HAT) agent, which is then trapped by benzoyl chloride. Once again, the authors used a bespoke photocatalyst, Cz‐NI‐Ph, that was not available to us or included in the ML model and was therefore not tested. As shown in Figure 6, the algorithm gave a high confidence rating for **4CzIPN** and **Ru1**, while also suggesting **RoB**, [Ru(phen)_3_](PF_6_)_2_ (**Ru2**), and **EY**, albeit with much lower confidence ratings. All five proposed photocatalysts are viable, with **RoB** affording a high yield of 70%. We also tested **Ir1** and **Ir3** as the human researcher choices, both of which also performed well; in fact, **Ir1** gave a comparable yield to **RoB** of 71%. However, it is important to note that the principal photochemistry in this reaction is being performed by pentafluoronitrobenzene, acting as a strong electron acceptor and proposed HAT agent that is compatible with all of the photocatalysts, of which the ML has no knowledge or mechanistic insight. From the perspective of reaction development, the current ML model therefore has limited value, as there is no included thermodynamic discriminator in terms of the optoelectronic properties of the photocatalysts, nor an understanding of the mechanistic importance of co‐additives that are needed for realizing the reaction. However, the ML model did identify **RoB** as a competitive photocatalyst, one that is typically not included in photocatalyst screening in the literature, and it performed exceptionally well in this reaction, on par with **Ir1**.

### Aldehyde to Nitrile Conversion

**Figure 7:**
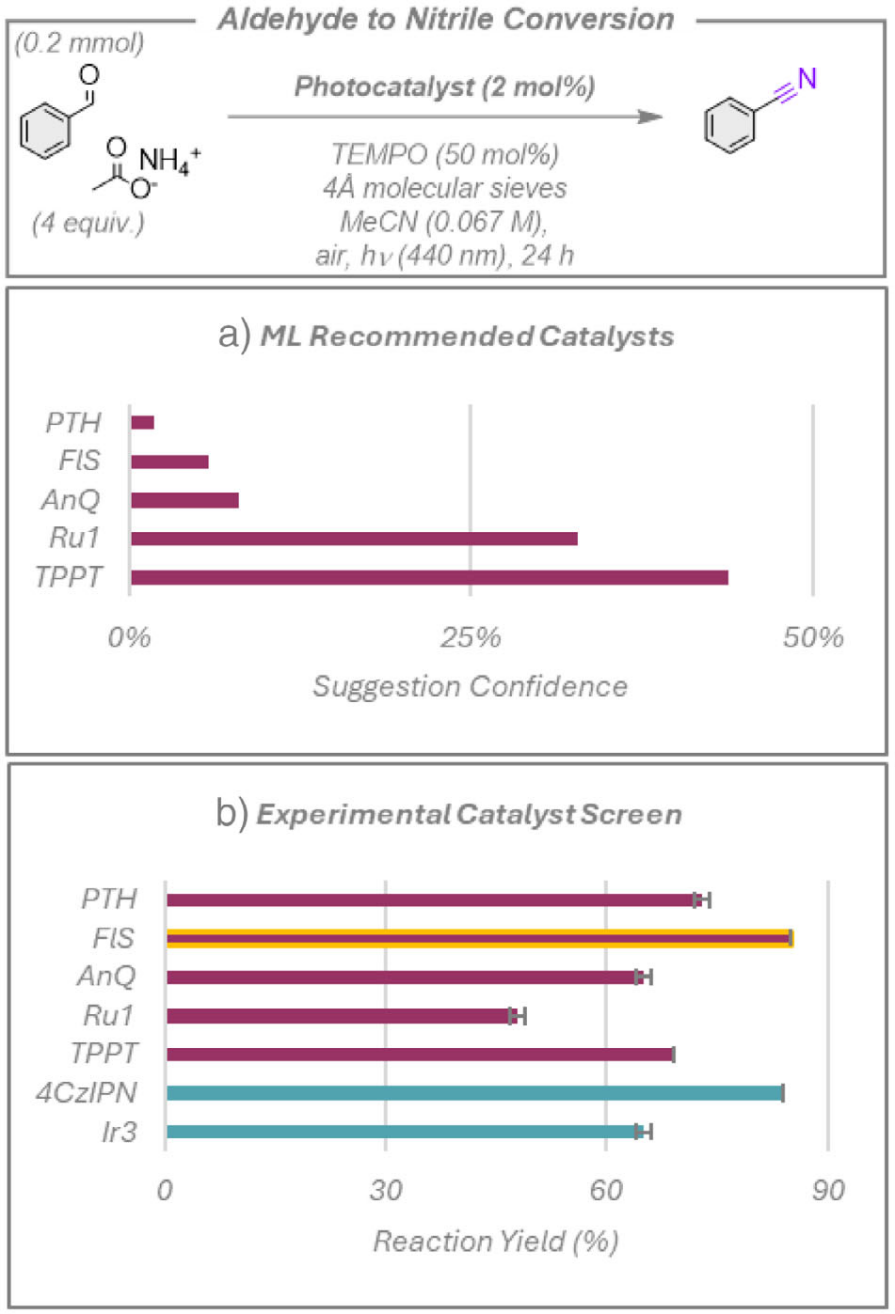
Photocatalyzed aldehyde to nitrile conversion. a) Top five photocatalysts suggested by the model and ranked by the confidence rating. b) Experimental yields. Red bars show the photocatalysts suggested by the model, with the yellow outline highlighting a previously untested yet highly productive photocatalyst, **FlS**, which the ML model identified. Blue bars represent additional photocatalysts chosen by human researchers. Yields presented are the average of two separate quantitative ^1^H NMR experiment repeats, using 1,3,5‐trimethoxybenzene as an internal standard, with the error bars indicating standard deviations.

The final electron transfer reaction explored was the synthesis of benzonitrile from benzaldehyde using TEMPO as an organic co‐catalyst, Figure 7.^[^ ^42^ ^]^ For this reaction, the original publication identified **4CzIPN** as the optimal photocatalyst during their photocatalyst screen. The top two photocatalysts recommended by the ML model (see Figure 7), **Ru1** and 2,4,6‐triphenylpyrylium tetrafluoroborate (**TPPT**), performed well (48% and 69%, respectively), but returned somewhat lower yields than **4CzIPN** (84%). The model's suggestions of anthraquinone (**AnQ**) and 10‐phenylphenothiazine (**PTH**) also gave comparably high yields of 65 and 73%, respectively. However, the model's suggestion of fluorescein (**FlS**), which the authors notably did not screen during the reaction development, gave comparable yields to **4CzIPN** (85%). This is a remarkable outcome for the model, as **FlS** is significantly cheaper than **4CzIPN**. However, we should again note that the reaction is driven primarily by the TEMPO co‐catalyst, of which the ML has no knowledge and does not factor into its decision‐making algorithm. While this means the model could not have been used to develop the initial reaction protocol during the reaction discovery phase, it clearly has demonstrated value during the optimization phase to identify superior photocatalysts for this transformation.

### Energy Transfer Example

**Figure 8:**
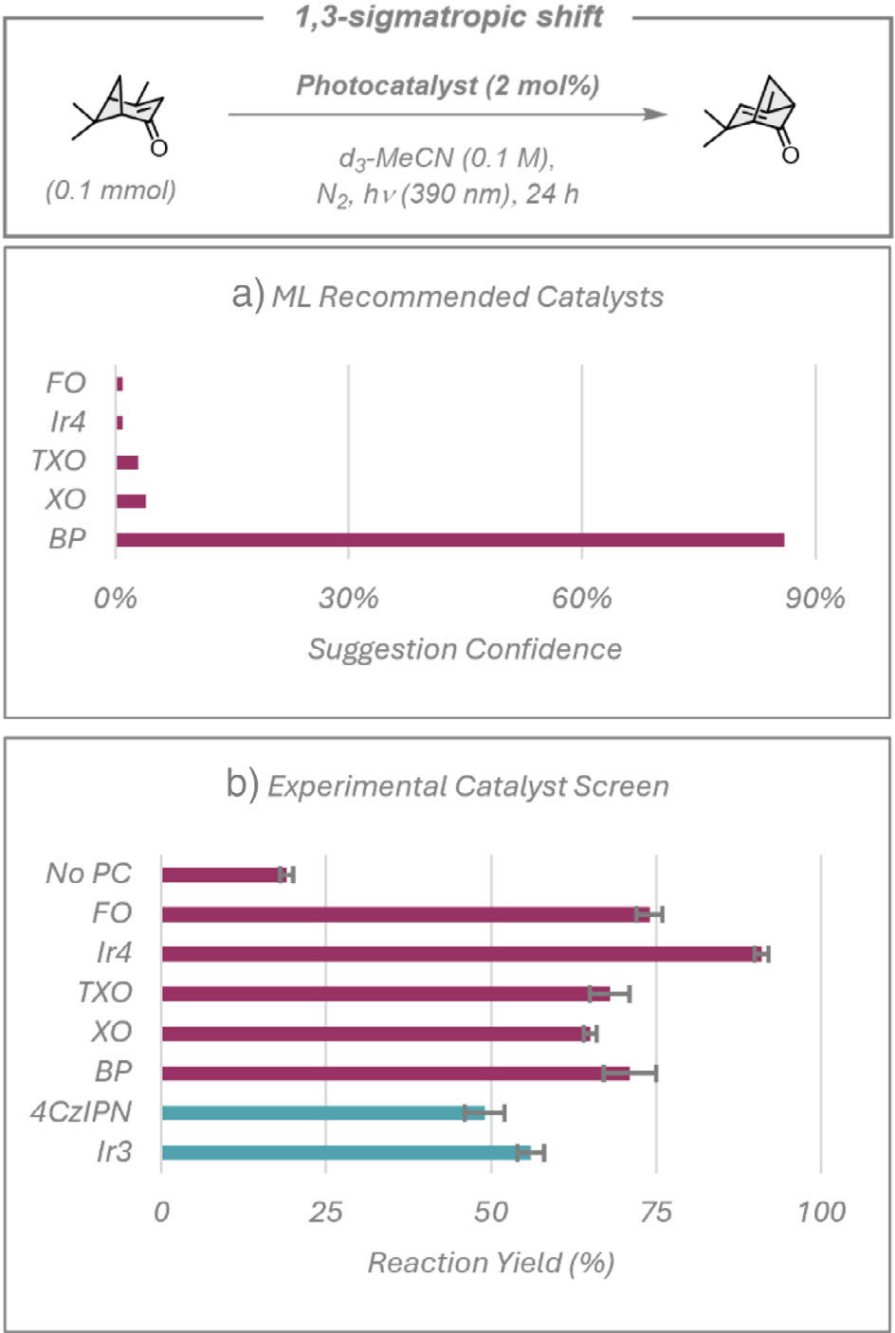
[1,3]‐sigmatropic shift of (S)‐verbenone to chrysanthenone. a) Top five photocatalysts suggested by the model and ranked by confidence rating. b) Experimental yields. Red bars show the catalysts suggested by the model (and a “No PC” control without photocatalyst). Blue bars represent additional photocatalysts chosen by human researchers. Yields presented are the average of two separate quantitative ^1^H NMR experiment repeats, using mesitylene as an internal standard, with the error bars indicating the standard deviation.

Finally, we examined the performance of the ML model in a photoinduced energy transfer reaction, the [1,3]‐sigmatropic shift of (S)‐verbenone to chrysanthenone, Figure 8.^[^ ^43^ ^]^ For this transformation, the ML model suggested predominantly organic diarylketone‐based photosensitisers, including benzophenone (BP), xanthone (XO), thioxanthone (TXO), and 9‐fluorenone (FO), as well as suggesting *fac*‐Ir(dFppy)_3_ (Ir4). Due to the onset of the absorbance spectra of the diarylketone photocatalysts, a 390 nm light source was used to drive the reaction (at this excitation wavelength, an inefficient, 19% yield reaction is observed in the absence of any catalysts). Once again, 4CzIPN and Ir3 were chosen as the human researcher choices to test alongside the model's suggestions, and both produced moderate yields of 49% and 56%, respectively. All of the model's suggested photocatalysts outperformed these “go‐to” examples, with the diarylketone‐based ones achieving 65%–74% yield, while Ir4 afforded an excellent 91% yield. Diarylketone‐based photocatalysts are known to undergo deactivation via pinacol homo‐coupling,^[^ ^44^ ^]^ which may explain why they performed less efficiently than Ir4 at the 2 mol% catalyst loading used for this reaction. In a broader context, because of the high triplet energy of the starting material (*E*_T_ = 3.0 eV),^[^ ^43^ ^]^ it could be expected that photocatalysts possessing similarly high *E*_T_ should perform more efficiently in the reaction than those whose *E*_T_ was much lower in energy. Therefore, it is not surprising that 4CzIPN and Ir3 (*E*_T_ = 2.53 and 2.68 eV, respectively) were less efficient photocatalysts than those suggested by the ML model (*E*_T_ = 2.75, 3.00, 3.22, and 2.84 eV for Ir4, BP, XO, and TXO, respectively).^[^ ^45^ ^]^ What is surprising, however, is that the model—despite having no prior knowledge of the *E*_T_ of either the photocatalysts or substrates—exclusively suggested photocatalysts that were more likely to facilitate favorable Dexter energy transfer to the substrate. While an experienced photochemist likely would have predicted this result, a researcher who has more limited experience in this field may have unnecessarily devoted resources to testing catalysts such as 4CzIPN and Ir3 as part of a photocatalyst screening campaign.

## Conclusions

The conclusions derived from these experiments are nuanced. In all of the reactions screened, every photocatalyst suggested by the ML model was productive, although rankings within each set of suggested photocatalysts do not correlate with experimental yields and cannot be used to infer which photocatalyst is optimal. ML‐suggested photocatalysts afforded yields that were consistently better than or on par with the photocatalysts chosen for a particular reaction by human researchers who selected only popular photocatalysts such as **4CzIPN** or **Ir3** in four out of the five reactions tested. We have also compared the outputs of the ML model to three other complex reaction systems from the recent literature, where the ML model selected similar photocatalysts to those chosen by expert photochemists during their screening programs (see Supporting Information for this discussion). From a practical point of view, one of the main advantages of the recommender is that it is not biased toward particular photocatalysts that human researchers consider “worth screening.” Here, the algorithm frequently suggested less popular photocatalysts that performed the reactions comparably well to the more widely used and more expensive “go‐to” photocatalysts (see Figure S1). For example, in the ATRA reaction, the original publication did not screen **Ru1**, which was suggested by the recommender, and this was, in fact, the best‐performing photocatalyst. In the dealkylative acylation, the ML model suggested **RoB** (albeit with a low confidence rating), and this turned out to be one of the best‐performing photocatalysts; notably, this is typically no longer a commonly screened photocatalyst. Similarly, in the aldehyde to nitrile conversion reaction, **FlS** performed as well as the much more popular **4CzIPN**.

While the ML model shows considerable promise as a tool to rapidly suggest photocatalysts, in its current form, it is not suited toward reaction discovery, where the selection of co‐catalysts, additives, solvents, and other reaction parameters is often more important than the choice of photocatalyst in optimizing the reaction yield, and this is a current limitation of the model; furthermore, there is no correlation between the confidence rating from the model and reaction yields. Overall, we suggest that in its current form, the recommender acts as an essential “sense check” tool to suggest the most promising photocatalysts to include in a photocatalyst screen, and we suggest that all photocatalysts recommended by the ML model be tested. Future versions of this ML tool will include thermodynamic and kinetic parameters of both photocatalysts and substrates, as well as excitation wavelengths, which will lead to stronger correlations between the confidence rating of the recommendation and the product yield. The model would also benefit from interrogating a more expansive photocatalyst library beyond the original 31 selected and an expanded library of reactions for each photocatalyst. Nonetheless, the ML model presented here may already act as a valuable tool for photocatalysis researchers.

## Supporting Information

Supporting Information provides additional theoretical and experimental details. All model code and data is now made available at: https://github.com/GlockPL/Photocatalyst‐Recommender‐based‐on‐a‐Large‐Language‐Model .

The research data supporting this publication can be accessed at https://doi.org/10.17630/21981110‐a42c‐44cd‐91aa‐b5c52a60a7b7.

## Author Contributions

M.K. and S.S. curated the data and developed the recommender models. F.M. co‐wrote the manuscript and directed the experimental validation efforts. J. B.‐S. and R.S. each conducted screening for two of the reactions. A.K.S. conducted screening for one reaction. V.M. scouted potential reactions. M.G. contributed to experimental data analysis, initial data set sorting, and designed the Table of Contents. M.B., T.C., L.H., A.M. contributed to initial data set sorting. B.A.G. and E.Z.‐C. conceived and supervised the project, and co‐wrote the manuscript.

## Conflict of Interests

The authors declare no conflict of interest.

## Supporting information



Supporting Information

Supporting Information

## Data Availability

The data that support the findings of this study are openly available in Pure at https://doi.org/10.17630/21981110‐a42c‐44cd‐91aa‐b5c52a60a7b7, reference number 321347017.
