# Supplementary material for: Design and Experimental Validation of a Photocatalyst Recommender Based on a Large Language Model
Source: Angew Chem Int Ed Engl. 2025 Dec 9;65(4):e14544. doi: 10.1002/anie.202514544 (PMC12828469; doi:10.1002/anie.202514544)
Supplement: Supplementary file 1 — Supporting Information [file ANIE-65-e14544-s001.docx]

Supplementary Materials

Design and Experimental Validation of a Photocatalyst Recommender based on a Large Language Model

Francis Millward,^ǂ[a]^ Michał Kulczykowski,^ǂ[b,c]^ Jay Badland-Shaw,^[a]^ Sara Szymkuć,^[b]^ Rajan Suraksha,^[a]^ Aniket Kumar Srivastawa,^[a]^ Violaine Manet,^[a]^ Máire Griffin,^[a]^ Megan Bryden,^[a]^ Thomas Comerford,^[a]^ Lea Hämmerling,^[a]^ Aminata Mariko,^[a]^ Bartosz A. Grzybowski,*^[d]^ Eli Zysman-Colman*^[a]^

[a] J. Badland-Shaw, Dr. M. Bryden, Dr. T. Comerford, M. Griffin, Dr. L. Hämmerling, V. Manet, A. Mariko, F. Millward, R. Suraksha , A. Kumar Srivastawa, Prof. Dr. E. Zysman-Colman

Organic Semiconductor Centre, EaStCHEM School of Chemistry, University of St Andrews; St Andrews, KY16 9ST, United Kingdom.
E-mail: eli.zysman-colman@st-andrews.ac.uk

[b] Dr. M. Kulczykowski, Dr. Sara Szymkuć
Allchemy, Inc., Highland, 46322 IN (USA)

[c] Dr. M. Kulczykowski

Institute of Organic Chemistry, Polish Academy of Science, ul. Kasprzaka 44/52, 02-224, Warsaw, Poland

[d] Prof. Dr. B. A. Grzybowski
Center for Algorithmic and Robotized Synthesis (CARS) of Korea’s Institute for Basic Science (IBS) and Department of Chemistry,
Ulsan National Institute of Science and Technology 50,
UNIST-gil, Eonyang-eup, Ulju-gun, Ulsan (South Korea)
E-mail: nanogrzybowski@gmail.com

**Data Preparation**

Each photocatalyst was queried in Reaxys using a set of keywords corresponding to its different synonyms. At this stage counterions, if present, were omitted. After downloading the raw data, the following filtering steps were performed:

(i) Initial filtering: entries with a null reaction field were removed. The remaining entries were checked if both substrates and products were present. Entries lacking either product or substrates structures were rejected.

(ii) Contextual filtering: at this second stage, the reaction conditions were checked for additional keywords indicating a photocatalytic transformation. Additionally, to discern photocatalytic reactions from MW irradiated chemical transformations, entries with ‘microwave irradiation’ as a keyword were excluded.

In the subsequent steps the reactions were mapped in order to deduce the atoms that’s change in the transformation and classify reactions according to its type. After this final step the curated dataset counted 36,097 reactions.

The Reaxys reaction IDs used in the building of this model are provided in the attached .csv file.

Table **S1**. List of photocatalysts employed included in the dataset.

| **Label** | **Name** | **SMILES of the photocatalyst** |
| --- | --- | --- |
| 1 | Ir(ppy)_2_(dtbbpy) | CC(C)(C)c1ccnc(-c2cc(C(C)(C)C)ccn2)c1.c1ccc(-c2ccccc2[Ir]c2ccccc2-c2ccccn2)nc1 |
| 2 | Ru(phen)_3_ | [Ru].c1cnc2c(c1)ccc1cccnc12.c1cnc2c(c1)ccc1cccnc12.c1cnc2c(c1)ccc1cccnc12 |
| 3 | Ir(ppy)_3_ | c1ccc(-c2ccccc2[Ir](c2ccccc2-c2ccccn2)c2ccccc2-c2ccccn2)nc1 |
| 4 | Ru(bpy)_3_ | [Ru].c1ccc(-c2ccccn2)nc1.c1ccc(-c2ccccn2)nc1.c1ccc(-c2ccccn2)nc1 |
| 5 | Cu(dap_2_) | COc1ccc(-c2ccc3ccc4ccc(-c5ccc(OC)cc5)nc4c3n2)cc1.COc1ccc(-c2ccc3ccc4ccc(-c5ccc(OC)cc5)nc4c3n2)cc1.[Cu+] |
| 6 | Ir(dfppy)_3_ | Fc1ccc(-c2ccccn2)c(F)c1.Fc1ccc(-c2ccccn2)c(F)c1.Fc1ccc(-c2ccccn2)c(F)c1.[Ir] |
| 7 | Ir(dF(CF_3_)ppy)_2_ (dtbbpy) | CC(C)(C)c1ccnc(-c2cc(C(C)(C)C)ccn2)c1.Fc1ccc(-c2ccc(C(F)(F)F)cn2)c(F)c1.Fc1ccc(-c2ccc(C(F)(F)F)cn2)c(F)c1.[Ir+] |
| 8 | Cu(dmphen)(Xantphos) | CC1(C)c2cccc(P(c3ccccc3)c3ccccc3)c2Oc2c(P(c3ccccc3)c3ccccc3)cccc21.Cc1ccc2ccc3ccc(C)nc3c2n1.[Cu+] |
| 9 | Ru(bpz)_3_ | [Ru].c1cnc(-c2cnccn2)cn1.c1cnc(-c2cnccn2)cn1.c1cnc(-c2cnccn2)cn1 |
| 10 | Ru(dtbbpy)_3_ | CC(C)(C)c1ccnc(-c2cc(C(C)(C)C)ccn2)c1.CC(C)(C)c1ccnc(-c2cc(C(C)(C)C)ccn2)c1.CC(C)(C)c1ccnc(-c2cc(C(C)(C)C)ccn2)c1.[Ru] |
| 11 | 4DPAIPN | N#Cc1c(N(c2ccccc2)c2ccccc2)c(C#N)c(N(c2ccccc2)c2ccccc2)c(N(c2ccccc2)c2ccccc2)c1N(c1ccccc1)c1ccccc1 |
| 12 | Anthraquinone | O=C1c2ccccc2C(=O)c2ccccc21 |
| 13 | Rhodamine B | CCN(CC)c1ccc2c(-c3ccccc3C(=O)O)c3ccc(=[N+](CC)CC)cc-3oc2c1 |
| 14 | TPPT | c1ccc(-c2cc(-c3ccccc3)[o+]c(-c3ccccc3)c2)cc1 |
| 15 | Tetrachloro-1,4-benzoquinone | O=C1C(Cl)=C(Cl)C(=O)C(Cl)=C1Cl |
| 16 | Fluorenone | O=C1c2ccccc2-c2ccccc21 |
| 17 | 9,10-dicyanoanthracene | N#Cc1c2ccccc2c(C#N)c2ccccc12 |
| 18 | 4CzIPN | N#Cc1c(-n2c3ccccc3c3ccccc32)c(C#N)c(-n2c3ccccc3c3ccccc32)c(-n2c3ccccc3c3ccccc32)c1-n1c2ccccc2c2ccccc21 |
| 19 | 10-phenylphenothiazine | c1ccc(N2c3ccccc3Sc3ccccc32)cc1 |
| 20 | Fluorescein | O=C1OC2(c3ccc(O)cc3Oc3cc(O)ccc32)c2ccccc21 |
| 21 | 1,4-dicyanonapthalene | N#Cc1ccc(C#N)c2ccccc12 |
| 22 | Rose bengal | O=C([O-])c1c(Cl)c(Cl)c(Cl)c(Cl)c1-c1c2cc(I)c(=O)c(I)c-2oc2c(I)c([O-])c(I)cc12 |
| 23 | Methylene Blue | CN(C)c1ccc2nc3ccc(=[N+](C)C)cc-3sc2c1 |
| 24 | Michler’s ketone | CN(C)c1ccc(C(=O)c2ccc(N(C)C)cc2)cc1 |
| 25 | Thioxanthone | O=c1c2ccccc2sc2ccccc12 |
| 26 | Mes-Acr | Cc1cc(C)c(-c2c3ccccc3[n+](C)c3ccccc23)c(C)c1 |
| 27 | Xanthone | O=c1c2ccccc2oc2ccccc12 |
| 28 | Benzophenone | O=C(c1ccccc1)c1ccccc1 |
| 29 | 1,4-dicyanobenzene | N#Cc1ccc(C#N)cc1 |
| 30 | Rhodamine 6G | CCNc1cc2oc3cc(=[NH+]CC)c(C)cc-3c(-c3ccccc3C(=O)OCC)c2cc1C |
| 31 | Eosin Y | O=C(O)c1ccccc1-c1c2cc(Br)c(=O)c(Br)c-2oc2c(Br)c(O)c(Br)cc12 |

Table **S2**. SMILES of the reactions tested for the experimental validation.

| Reaction 1a (*ATRA*) | ClS(C1=CC=CC=C1)(=O)=O.C=CC[B-](F)(F)F>>O=S(C2=CC=CC=C2)(CC=C)=O |
| --- | --- |
| Reaction 1b (*ATRA*) | ClS(C1=CC=C(C)C=C1)(=O)=O.C=CC[B-](F)(F)F>>O=S(C2=CC=C(C)C=C2)(CC=C)=O |
| Reaction 1c (*ATRA*) | ClS(C1=CC=C(C(F)(F)F)C=C1)(=O)=O.C=CC[B-](F)(F)F>>O=S(C2=CC=C(C(F)(F)F)C=C2)(CC=C)=O |
| Reaction 2 (*Phosphorylation*) | O=P(C1=CC=CC=C1)C2=CC=CC=C2.CCN(CC)CC>>O=P(C3=CC=CC=C3)(C(C)N(CC)CC)C4=CC=CC=C4 |
| Reaction 3 (Acylation) | CN(C)C1=CC=CC=C1.ClC(C2=CC=CC=C2)=O>>O=C(N(C)C3=CC=CC=C3)C4=CC=CC=C4 |
| Reaction 4 (*Aldehyde to nitrile interconversion*) | O=CC1=CC=CC=C1.CC([O-])=O.[H][N+]([H])([H])[H]>>N#CC2=CC=CC=C2 |
| Reaction 5 (*1,3-sigmatropic shift*) | O=C1C2CC(C2(C)C)C(C)=C1>>O=C3C4CC=C(C)C3C4(C)C |


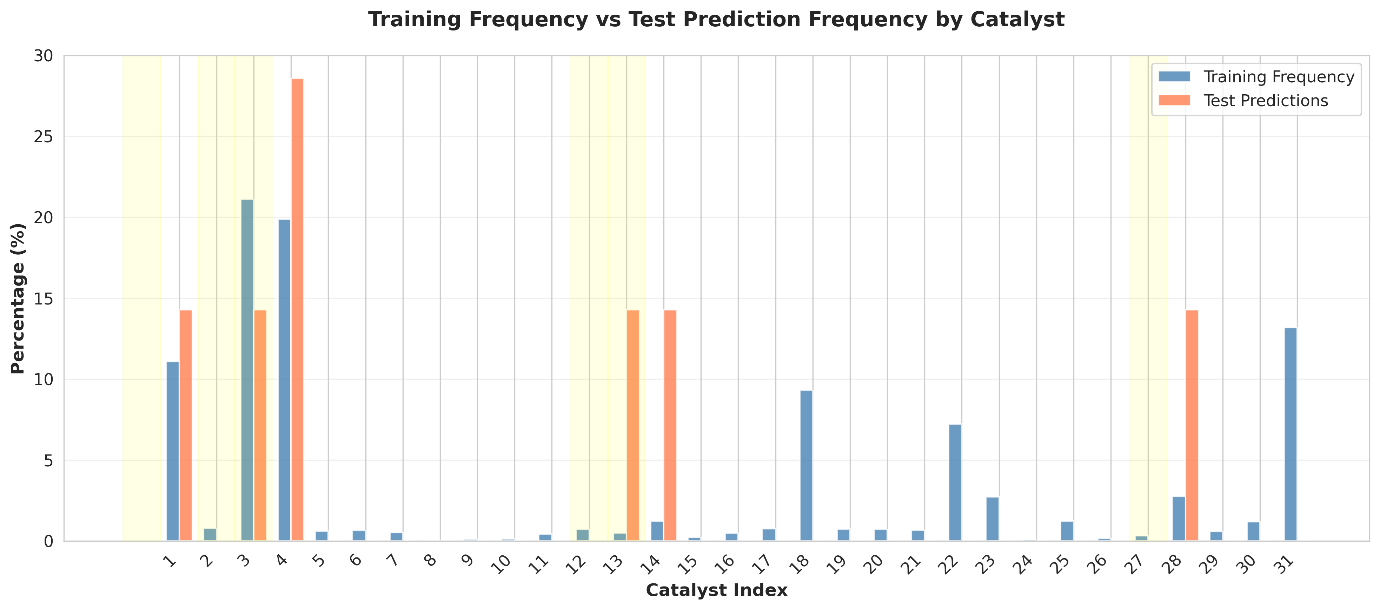


Figure **S1**. Popularity of catalysts in the training and validation sets. Blue bars represent how often a given catalyst was encountered in the training reaction data; the orange bars plot the frequencies of the catalysts in the validation dataset (i.e., those committed to experimental validation). As seen, whereas for the popular photocatalysts #1, #3 and #4, a strong correlation observed, there is no such correlation for photocatalysts #13, #14 and #28. In fact, the overall Spearman correlation coefficient between the training and test sets is modest, r = 0.47 with p = 0.007.


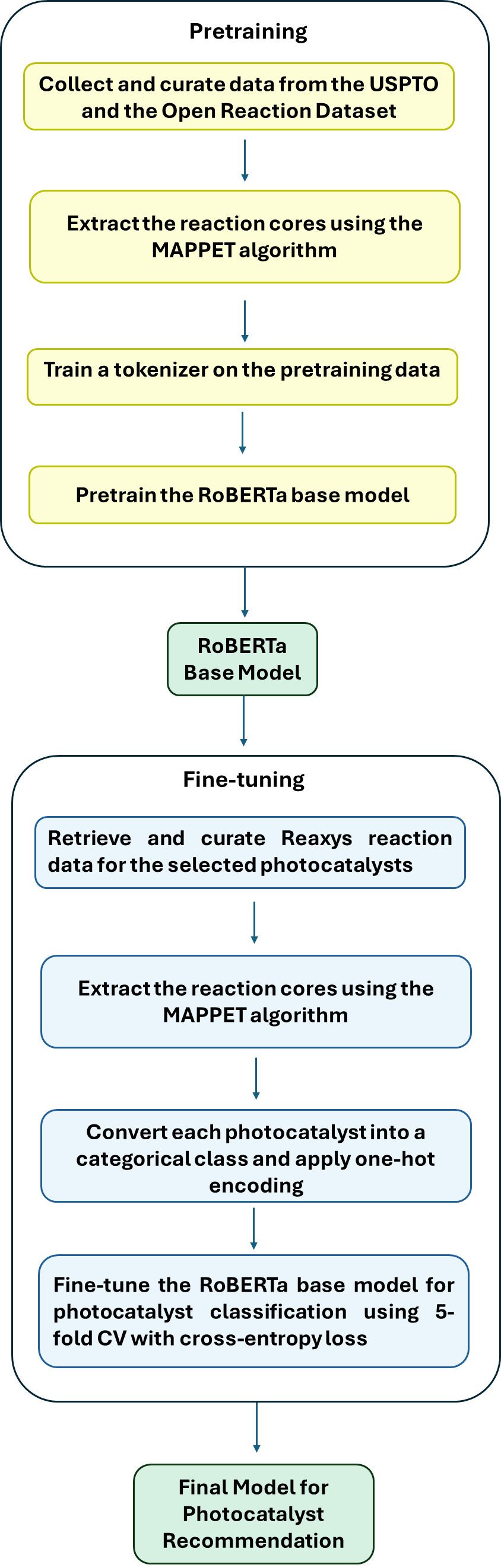


**Figure S2.** The flowchart describing the training of the Photocatalyst Recommender Model. In the pretraining stage, reaction data are collected and curated from USPTO and ORD datasets. For each unique entry, a reaction core is extracted to capture the atoms that change their bonding patterns along with their local environments. These reaction templates, together with the corresponding reaction SMILES strings, are used to train the tokenizer that preserves meaningful structural patterns in SMILES strings. The resulting tokenized data enable pretraining of a RoBERTa model, allowing it to learn common rules and patterns associated with chemical reactivity. In the second stage, the model is adapted for photocatalyst recommendation. Representative 31 photocatalysts are selected, and reactions involving these catalysts are retrieved from the Reaxys database. Reaction templates are again extracted, and each photocatalyst is assigned to a categorical class. The model is then fine-tuned on this labelled dataset to classify suitable photocatalysts for new reactions.

**Reaction condition information available within the training set**

After assigning each entry a reaction group ID, the Reaxys data were further processed by de-duplicating entries with identical reaction SMILES, photocatalyst ID, and reaction group ID. This operation reduced the dataset from 43,686 to 36,097 entries. This de-duplicated set was evaluated for missing entries in Reaxys’ *Reaction-condition* columns. Because photocatalytic reactions are performed in solution, the *Solid phase* column is not applicable and was uniformly empty. Similarly, *Pressure* and *pH-Value* columns were also largely empty, expected for photocatalysis, in which pH is not typically applicable, and pressure is usually atmospheric and thus seldomly reported. Photocatalyst data were typically captured in the Reagent or Other Conditions columns. Regarding reaction media, 8.2% of the dataset (2,954 entries) lacked solvent information, 13.4% (4,922 entries) used solvent mixtures, and 78.4.% (28,221 entries) reported a single solvent. Within the single-solvent entries, 56 different solvents and one neat (solvent-free) conditions were reported. However, the dataset was quite unbalanced and dominated by only four solvents (Figure **S3**). The most popular ones were acetonitrile (10,590 entries, 37.5%), *N,N*-dimethylformamide (3,563 entries,12.6%), dimethyl sulfoxide (2,867 entries,10.2%) and dichloromethane (2,722 entries, 9.6%), together accounting for 69.9% of the single-solvent subset (19,742 entries across these four).


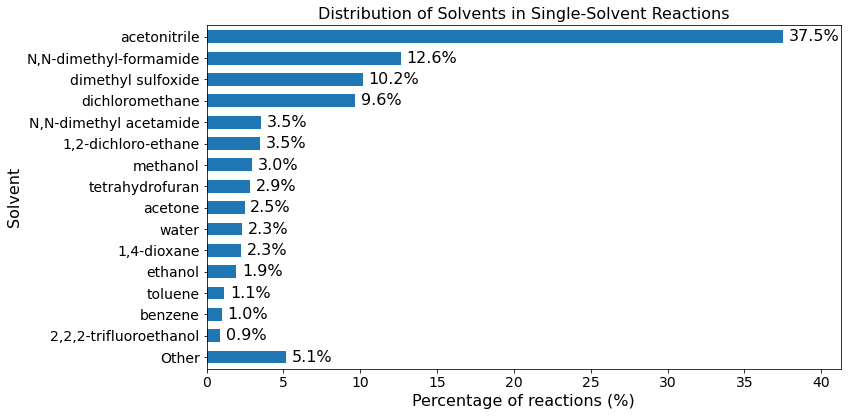


Figure **S3**. Histogram representing distribution of the single-solvent reactions in the training set. The top four solvents account for 69.9% of the set.

We also explored the presence of the additives, TEMPO and pentafluoronitrobenzene in the data set. For each additive, we created a dictionary of aliases to maximize the number of identified entries. Only 145 entries were identified, with TEMPO accounting for 81% (117 entries) and pentafluoronitrobenzene for the remaining 19% (28 entries). Such low counts make construction of any meaningful ML models problematic.

**Application of the model more complex reactions in the literature**

After completing the 5 validation experiments, we looked at the literature to see if the model could also suggest working photocatalysts for much more complicated reactions. For example, MacMillan and co-workers disclosed the cross-coupling of two alcohol species (*Science* **2024**, 383, 1350-1357). The coupling partners used in the optimization program were:


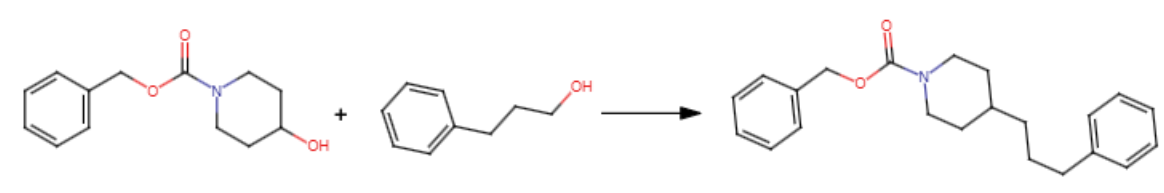


Feeding this reaction into the AI model, the following photocatalysts were suggested: [Ir(ppy)_2_(dtbbpy)]^+^, [Ru(bpy)_3_]^2+^, 4CzIPN, Eosin Y, *fac*-Ir(ppy)_3_. During their reaction optimisation, Macmillan and co-workers screened [Ir(ppy)_2_(dtbbpy)]^+^, [Ru(bpy)_3_]^2+^, 4CzIPN, and *fac*-Ir(ppy)_3_, giving yields of 27, 8, 49, and 11%, respectively. MacMillan only found one other photocatalyst that outperformed 4CzIPN; (which gave 54%).

Another example is the one-pot-two-step synthesis of semi-saturated polycyclic rings, also disclosed by MacMillan (*Nature* **2024**, 628, 326-332). The coupling partners used during the optimization program are shown here:


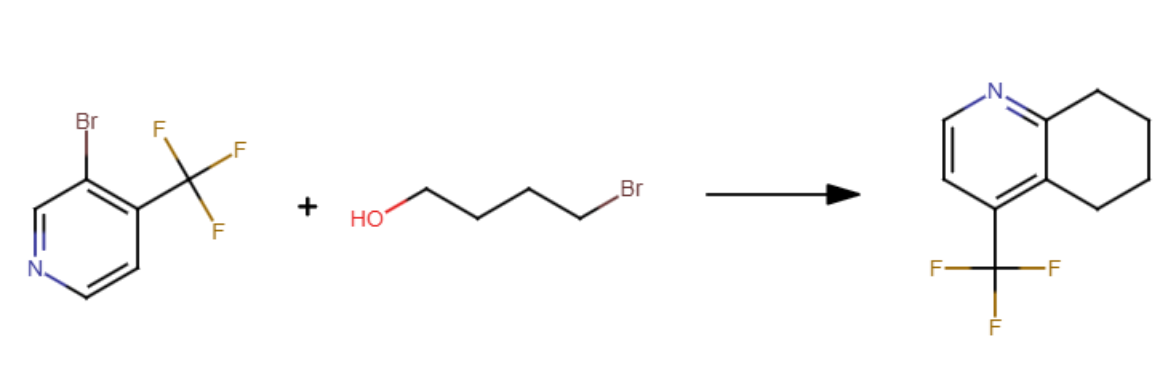


Our model suggested [Ir(ppy)_2_(dtbbpy)]^+^ with a confidence rating of 99%. MacMillan also tested this photocatalyst, which gave a yield of 29%, and was only beaten by a photocatalyst that was not in the database of the AI model ([Ir(FMeppy)_2_(dtbbpy)]^+^, which gave 49% yield).

A final example we tested was the generation of carbanions from alkenes (*Nature Catal*. **2024**, 7, 1316-1329). In this reaction, multiphoton absorptions and *in situ* catalyst modifications are implied, which further adds to the complexity of the system from a catalyst prediction standpoint. The coupling partners in the optimization of one of the reactions developed are shown here:


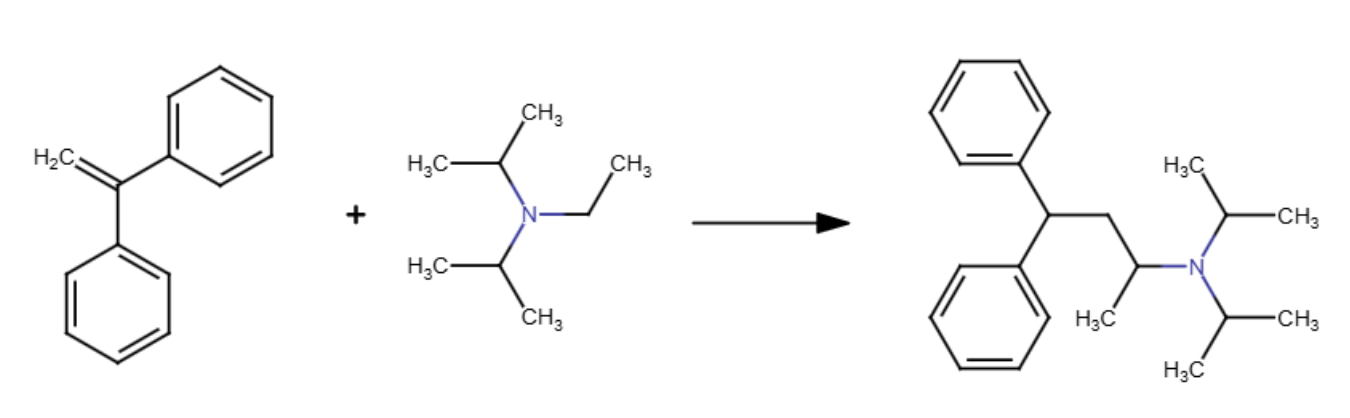


For this transformation, the AI model suggested [Ir(ppy)_2_(dtbbpy)]^+^, [Ru(bpy)_3_]^2+^, *fac*-Ir(ppy)_3_, dicyanobenzene, and 4CzIPN. In the reported supporting information, while [Ru(bpy)_3_]^2+^ was not able to facilitate the desired transformation, [Ir(ppy)_2_(dtbbpy)]^+^ and 4CzIPN worked, affording the product in 54 and 70% yields, respectively.

Thus, in all three of these highly complex reactions, working photocatalysts were selected by the recommender. This aligns well with the results from the experimental trials with the algorithm acting as an assistant that helps researchers not to miss potentially valuable photocatalysts when screening reactions.

**Additional studies on feature importance and feature space.**

Although chemical interpretability of transformer-based models is, in general, challenging – here, we wished to investigate whether at least some insights can be gained by the SHAP analysis and waterfall plots. In this example, we used this approach to study which input features contribute to differences in the confidence rating between sulfonyl chlorides bearing a trifluoromethyl versus a methyl group (example from the ATRA reaction in the main-text). The SHAP waterfall plots in **Figures S4** and **S5** deconstruct the model's top-ranked photocatalyst confidence score, with all contributions expressed in logit space (after application of the softmax function, these logits correspond to the final predicted probabilities of the assigned catalyst class). Starting from the dataset's average expected output *E[f(X)]* (base values = -1.155 and -0.018), the individual SHAP contributions accumulate sequentially to reach the final logit.

For the trifluoromethyl-bearing substrate, branching motifs such as “)“ and “(=“ as well as presence of oxygen-containing fragments provide the strongest positive contributions, although presence of fluorine atoms (F) is also amongst top-ten features. Interestingly for the methyl-bearing substrate, the presence of the “F” and “B” atoms dominates the list – in this case, however, these atoms come from the potassium allyltrifluoroborate substrate (used in both reactions considered in the main-text Figure 4). Also, the carbons “C” are not dominant but amongst the top features. Altogether, this means that while there are differences in the CH_3_ vs. CF_3_-containing substrates, it is difficult to ascribe model’s predictions to a particular functional group. The accompanying logit histograms (**Figures S6** and **S7**) show the scores across all catalyst classes, highlighting that a single logit must be interpreted in the context of the full distribution of outputs.


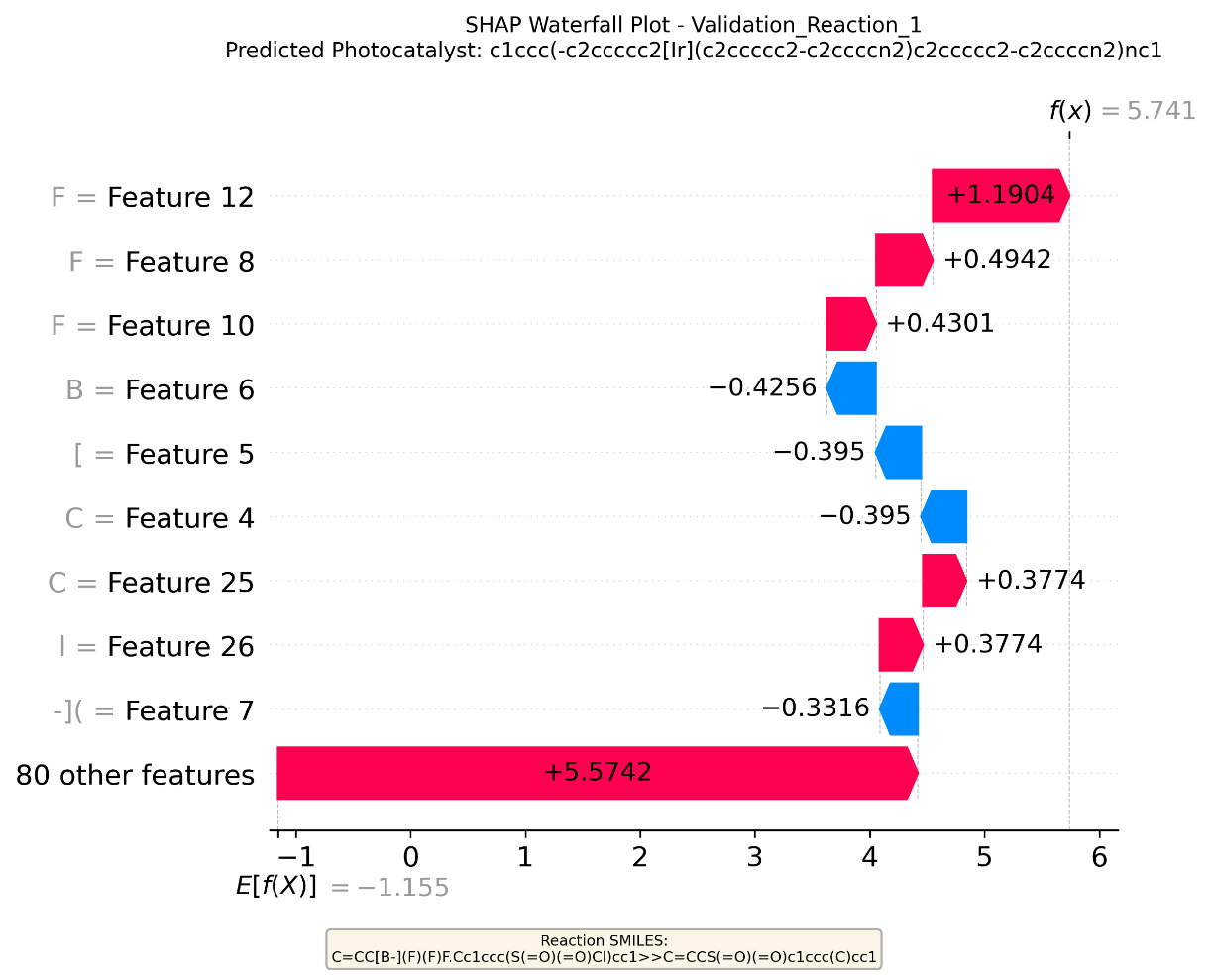
**Figure S4**. SHAP waterfall plot for the ATRA reaction with CH_3_-containing substrate.


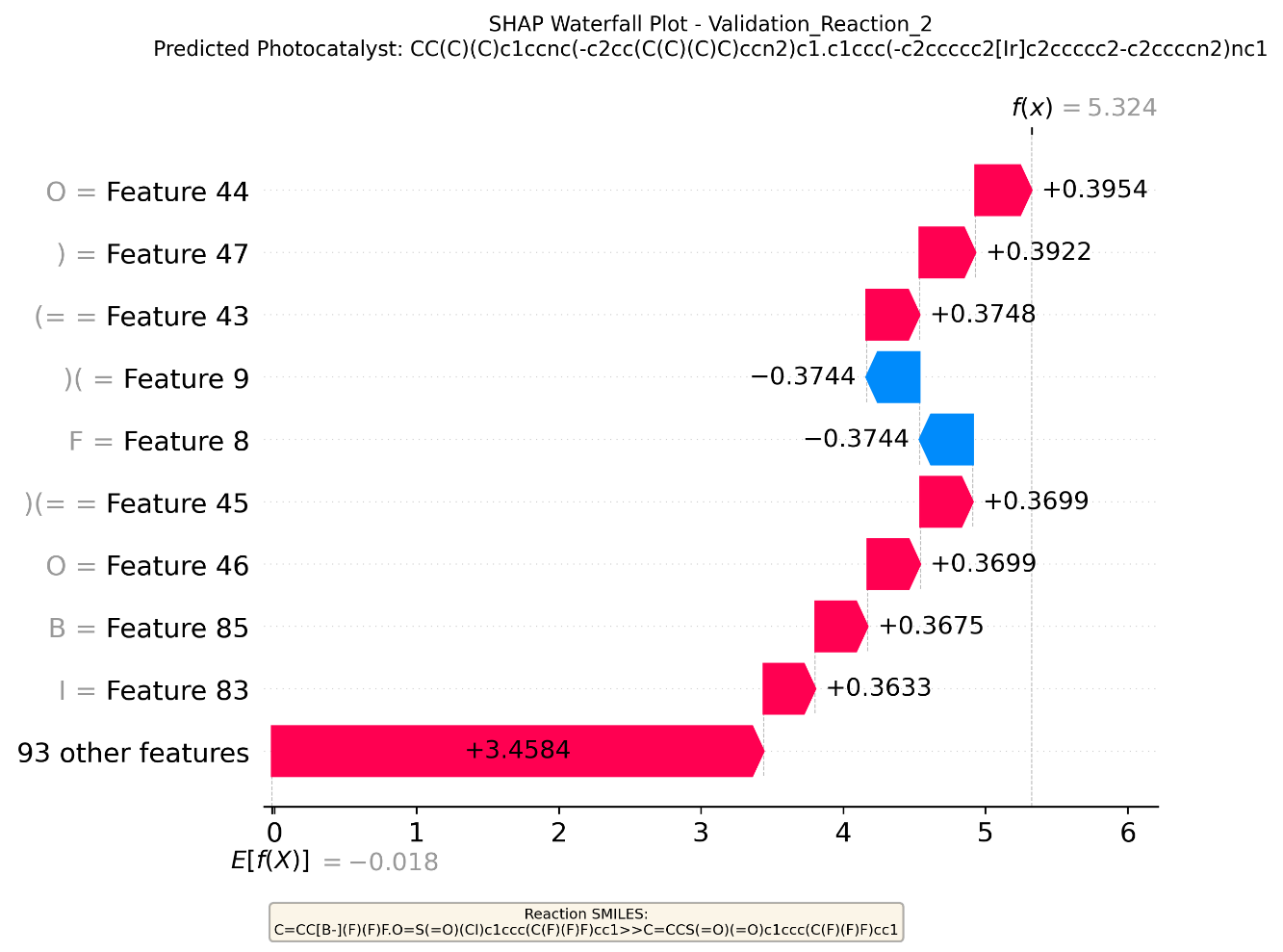


**Figure S5**. SHAP waterfall plot for the ATRA reaction with CF_3_-containing substrate.


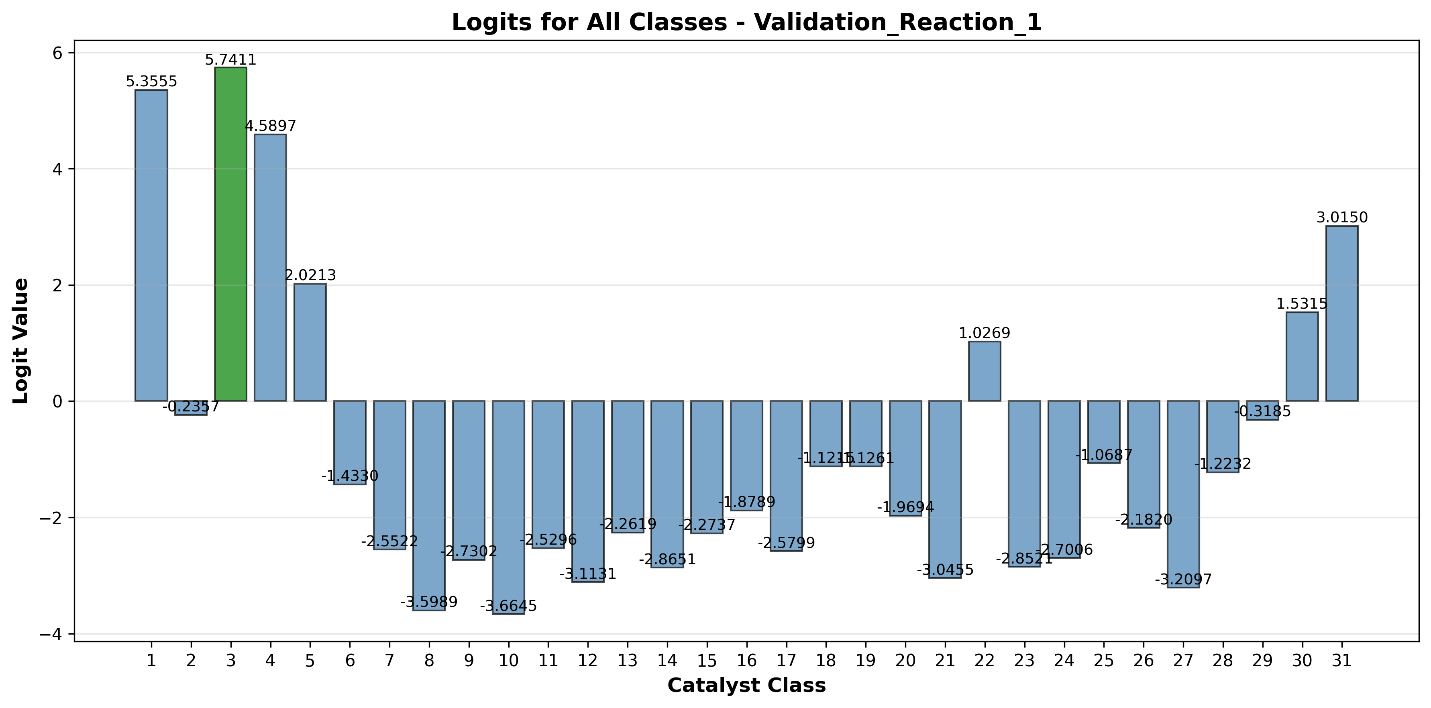


**Figure S6**. Logit histogram for the ATRA reaction with CH_3_-containing substrate.


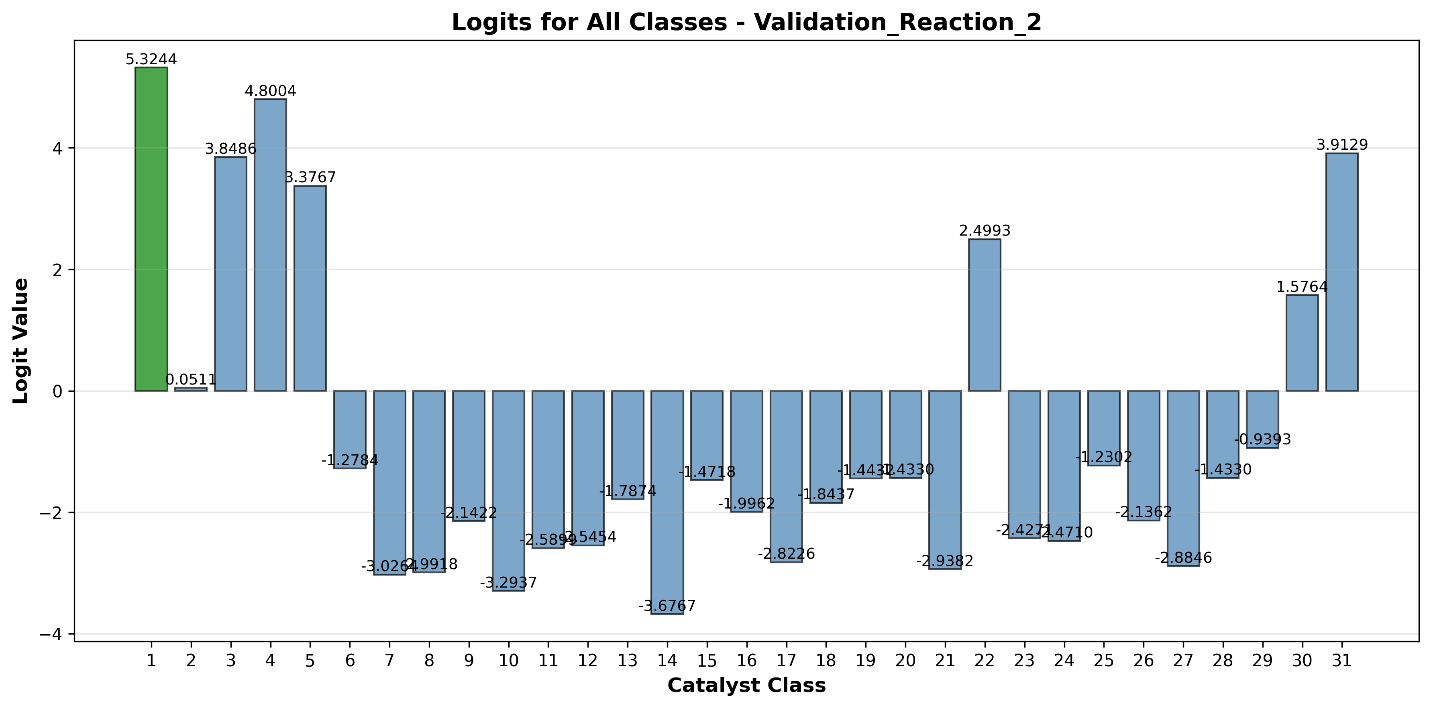


**Figure S7**. Logit histogram for the ATRA reaction with CF_3_-containing substrate.

We also visualized the test and validation reactions in the embeddings space by PCA, t-SNE and UMAP. These plots shown in **Figure S8** evidence that while the experimentally tested reactions are within distribution of the test set reactions, they probe it broadly (i.e., are not probing only a narrow region of the space).

*
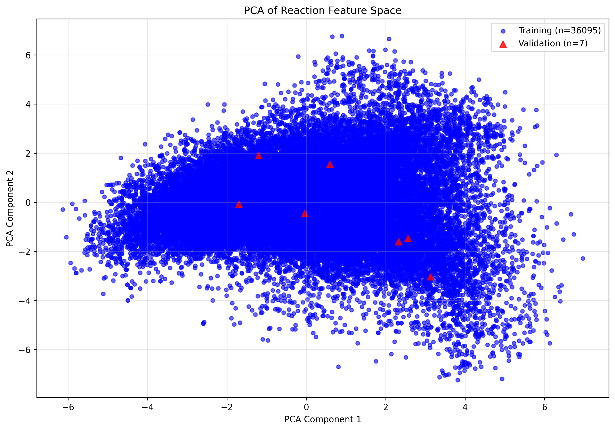
*


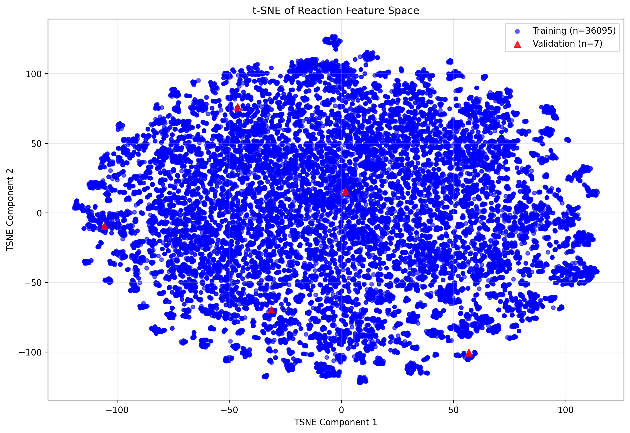


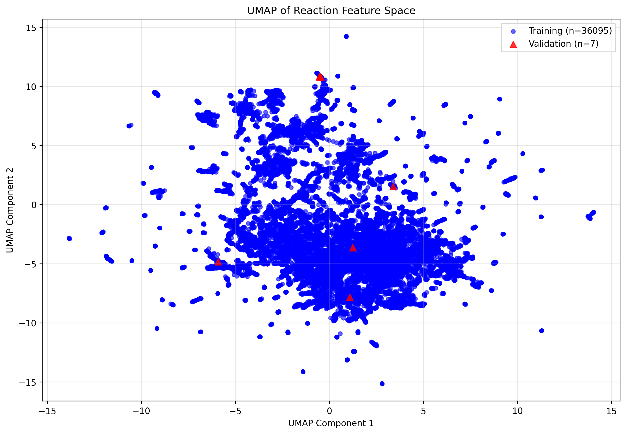


**Figure S8**. PCA, t-SNE and UMAP embeddings of the reactions in the training set (blue markers) and the experimental validation set (red markers).

**Tutorial for the Photocatalyst Recommender Web Application**

The web application is available at <https://photocatals.grzybowskigroup.pl/predict/> . In order to receive candidate photocatalysts from the model, it is necessary to provide a SMILES string representing the photocatalytic reaction in the following format:

Substrate1.Substrate2>>Product

After clicking the ‘PREDICT’ button, the model generates top five photocatalyst recommendations. Each candidate is presented as both a molecular structure and a SMILES string, along with the model’s confidence score.


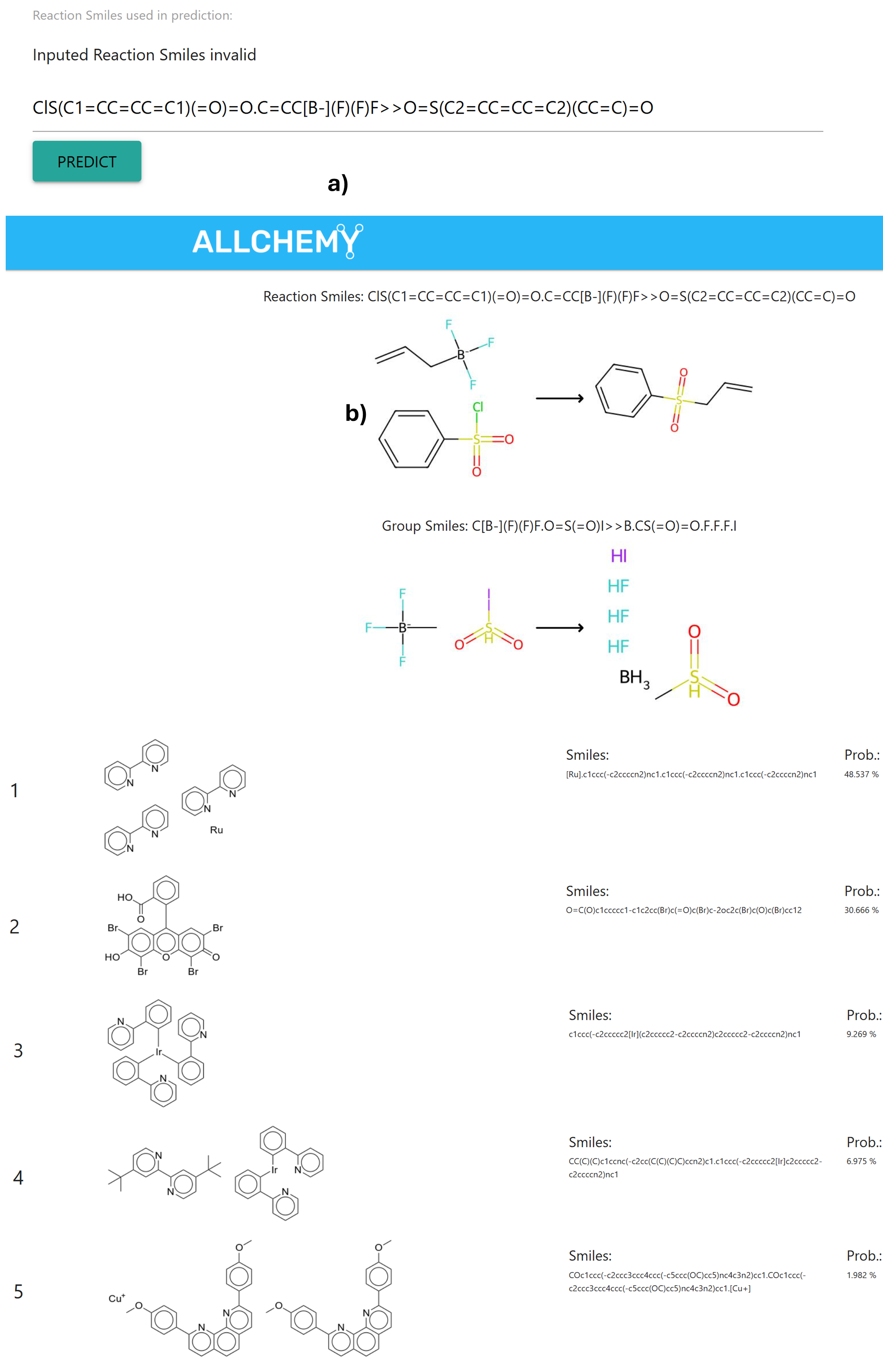


**Figure S9.** Overview of the Photocatalyst Recommender web application interface. Users can input a photocatalytic reaction in SMILES format (Substrate1.Substrate2>>Product) to obtain a ranked list of the top five predicted photocatalysts. The figure shows an example input (panel a) and the resulting recommendations, displayed as both molecular structures and SMILES strings, together with the model’s predicted probability scores (panel b).

Materials, Methods and Experimental Details

General synthetic procedures

The following catalysts and starting materials were synthesised according to literature procedures: *fac*-Ir(ppy)_3_,^1^ [Ir(ppy)_2_(dtbbpy)]PF_6_,^2^ [Ru(bpy)_3_][PF_6_]_2_ and [Ru(phen)_3_][PF_6_]_2_,^3^ [Ir(dF(CF_3_)ppy)_2_(dtbbpy)]PF_6_,^4^ 4CzIPN,^5^ [Cu(dap)_2_]Cl,^6^ [Cu(dmp)(xantphos)]PF_6_.^7^ All other catalysts, reagents and solvents were obtained from commercial suppliers and used as received. Air-sensitive reactions were performed under a nitrogen atmosphere using Schlenk techniques. Flash column chromatography was carried out using a CombiFlash NextGen automated column from Teledyne ISCO, with solid loading of samples using silica gel (Silia-P from Silicycle, 60 Å, 40-63 µm). Analytical thin-layer chromatography (TLC) was performed with silica plates with aluminium backings (250 µm with F-254 indicator). TLC visualization was accomplished by 254/365 nm UV lamp. NMR spectra were recorded on a Bruker Advance spectrometer (400 or 500 MHz for ^1^H). The following abbreviations have been used for multiplicity assignments: “s” for singlet, “d” for doublet, “t” for triplet, “q” for quartet, “m” for multiplet, “br” for broad, and qC for quaternary carbon. ^1^H spectra were referenced against residual solvent peaks with respect to TMS (δ = 0 ppm).

Details of the Solution-State Photoreactors

Two different photoreactors were used throughout the project.

Reactions 1 and 3 were conducted utilising a custom-built photoreactor inspired by the commercially available *HepatoChem PhotoRedOx Box™*, Figure **S10**.^8, 9^ *Kessil PR160L* LED lights were used as the excitation source, set at their maximum intensity for all experiments. A magnetic stirrer plate allows for continuous mixing of the reaction throughout the course of the reaction. 7 mL glass vials were used for all solution-state experiments. Fans in a push-pull configuration limit heating of reactions by LEDs.

Reactions 2, 4, and 5 were conducted utilising a custom-built photoreactor using a 3D printed vessel holder based on a design from Leonori and co-workers, Figure **S11**.^10^ The holder was then placed in a custom made photoreactor made from mirrored panels.^11^ A magnetic stirrer plate allows for continuous mixing throughout the course of the reaction. Fans in a push-pull configuration limit heating of reactions by LEDs. *Kessil PR160L* LED lights were used as the excitation source, set at their maximum intensity for all experiments. The LED is approximately 7-8 cm away from the reaction vials. The enclosed nature of the reactor limits light leakage and risk to researchers.


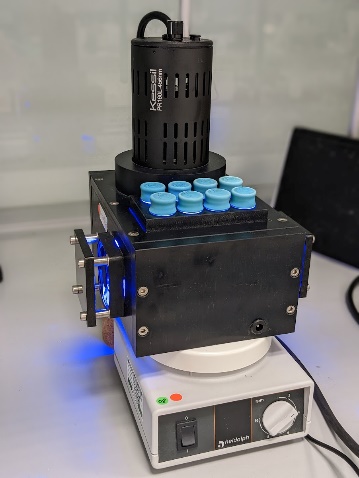


Figure S10. Solution-state photocatalysis reactor used for reactions 1 and 3.

**a)**


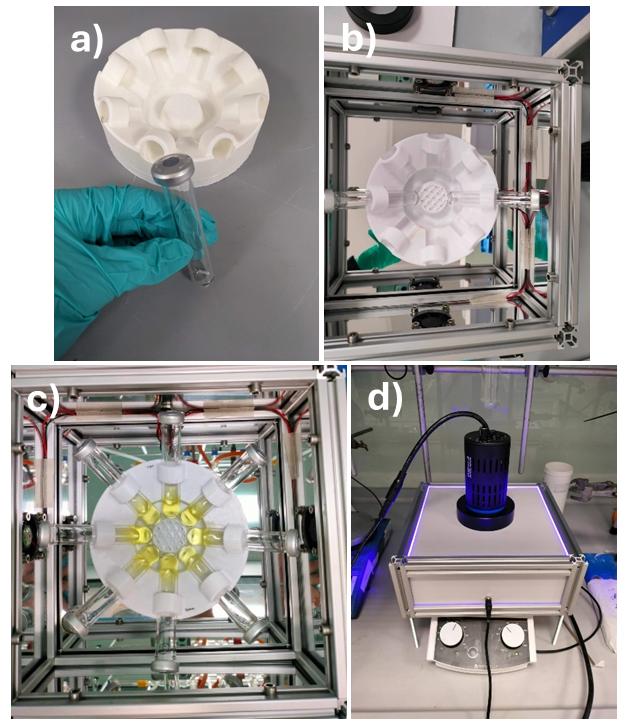


Figure **S11**. Solution-state photocatalysis reactor used for reactions 2, 4, and 5. a) Microwave vials with crimp lids and the 3D printed holder designed by Leonori and co-workers.^10^ b) Holder is placed inside a custom photoreactor; a mirrored box with 2 fans in a push pull configuration. c) Up to 8 reactions can be conducted inside the reactor simultaneously. d) The outside of the reactor with *Kessil* LED and stirrer plate.

***Reaction 1 (ATRA). Procedure adapted from Ref***  **^12^.** An oven-dried vial was charged with potassium allyltrifluoroborate (66.6 mg, 0.45 mmol, 1.5 equiv.), potassium carbonate (41.5 mg, 0.3 mmol, 1 equiv.), photocatalyst (1 mol%) and the sulfonyl chloride (if solid). The vial was degassed via sparging with nitrogen for 5 minutes. Dry acetonitrile (1 mL) and the sulfonyl chloride (if liquid) were added via syringe, and the reaction mixture was bubbled with nitrogen for 10 minutes. Sulfonyl chlorides used: benzenesulfonyl chloride (53.0 mg, 0.3 mmol, 1 equiv.), *p*-toluenesulfonyl chloride (57.2 mg, 0.3 mmol, 1 equiv.), or 4-(trifluoromethyl)benzenesulfonyl chloride (73.4 mg, 0.3 mmol, 1 equiv.). The reaction was irradiated with a 427 nm *Kessil* LED with stirring for 22 hours at room temperature. The reaction was diluted with brine:water (1:1, 40 mL) and washed with ethyl acetate (3 x 10 mL). The organic phase was dried over MgSO_4_, filtered, and the solvent removed under reduced pressure. The product yield was determined via quantitative ^1^H NMR spectroscopy using 1,3,5-trimethoxybenzene as an internal standard, with the product signals referenced against reported literature NMR spectroscopic data.^12^ Each reaction was repeated two times to check that results were reproducible.

***Reaction 2 (Phosphorylation). Procedure adapted from Ref*** ***^13^.*** An oven-dried vial was charged with diphenylphosphine oxide (20.2 mg, 0.1 mmol, 1 equiv.), triethylamine (41.8 μL, 0.3 mmol, 3 equiv.), photocatalyst (2.5 mol%), and dry MeCN (1 mL). The reaction was irradiated with 427 nm light from a *Kessil* LED with stirring for 22 hours at room temperature. The solvent was removed under reduced pressure, and the product yield was determined via quantitative ^1^H NMR spectroscopy using 1,3,5-trimethoxybenzene as an internal standard, with the product signals referenced against reported literature NMR spectroscopic data.^13^ Each reaction was repeated two times to check that results were reproducible.

***Reaction 3 (Acylation). Procedure adapted from Ref ^14^.*** An oven-dried vial was charged with potassium bicarbonate (10.0 mg, 0.1 mmol, 1 equiv.), *N,N*-dimethylaniline (12.1 mg, 0.1 mmol, 1 equiv.), pentafluoronitrobenzene (10.7 mg, 0.05 mmol, 0.5 equiv.), and the photocatalyst. The vial was degassed via sparging with nitrogen for 5 minutes before ethyl acetate (2 mL) and benzoyl chloride (15.5 mg, 0.1 mmol) were added via syringe. The reaction mixture was bubbled with nitrogen for 10 minutes, before being irradiated with a 427 nm *Kessil* LED with stirring for 22 hours at room temperature. The solvent was removed under reduced pressure, and the product yield was determined via quantitative ^1^H NMR spectroscopy using 1,4-(bistrimethylsilyl)benzene as an internal standard, with the product signals referenced against reported literature NMR spectroscopic data.^14^ Each reaction was repeated two times to check that results were reproducible.

***Reaction 4  (Aldehyde to nitrile interconversion). Procedure adapted from Ref*** ^15^***.*** An oven-dried vial was charged with ammonium acetate (61.7 mg, 0.8 mmol, 4 equiv.), photocatalyst (2 mol%), 3 Å molecular sieves (50 mg), benzaldehyde (21.2 mg, 0.2 mmol, 1 equiv.), and MeCN (3 mL). The reaction mixture was vigorously stirred for 30 minutes. The reaction was irradiated with a 440 nm *Kessil* LED with stirring for 24 hours at room temperature. The solvent removed was under reduced pressure, and the product yield was determined via quantitative ^1^H NMR spectroscopy using 1,3,5-trimethoxybenzene as an internal standard, with the product signals referenced against reported literature NMR spectroscopic data.^15^ Each reaction was repeated twice to verify that the results were reproducible.

***Reaction 5 (intramolecular [1,3] sigmatropic alkyl shift). Procedure adapted from Refs***^16, 17^**.** An oven-dried vial was charged with the photocatalyst (2 mol%). The vial was degassed via sparging with nitrogen for 5 minutes before deuterated acetonitrile (1 mL) was added via syringe. The reaction mixture was bubbled with nitrogen for 15 minutes. s-Verbenone (15 mg, 15 µL, 0.1 mmol, 1 equiv.) was added via syringe, and the reaction was bubbled with nitrogen for 1 minute. The reaction was stirred and irradiated using a 390 nm *Kessil* LED for 24 hours at room temperature. The product yield was determined via quantitative ^1^H NMR spectroscopy using mesitylene as an internal standard, with the product signals referenced against reported literature NMR spectroscopic data.^16, 17^ Each reaction was repeated twice to verify that the results were reproducible.

NMR Data

**
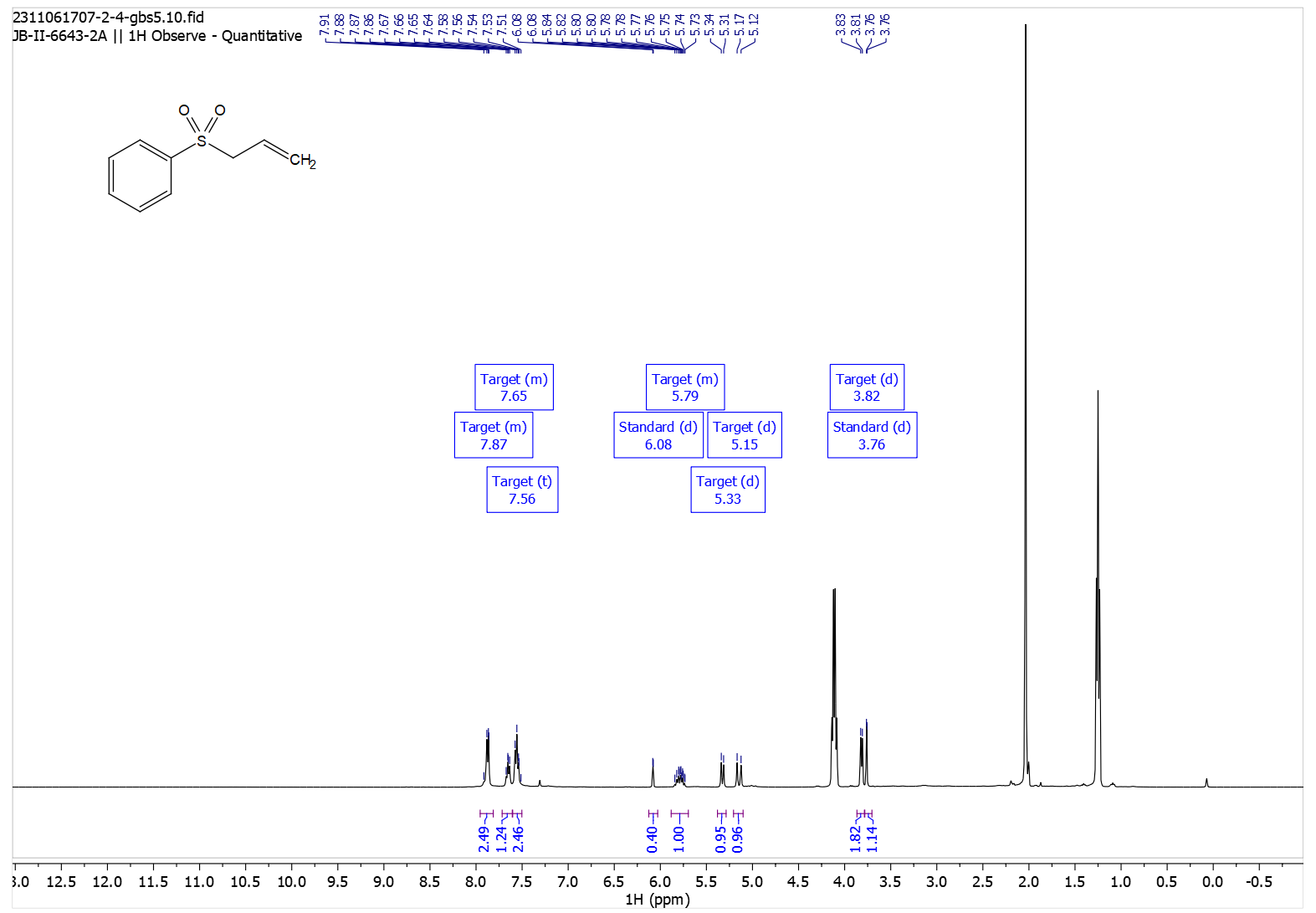
**

Figure **S12**. Example of ^1^H NMR spectrum of crude reaction mixture from Reaction 1a in CDCl_3_.

**
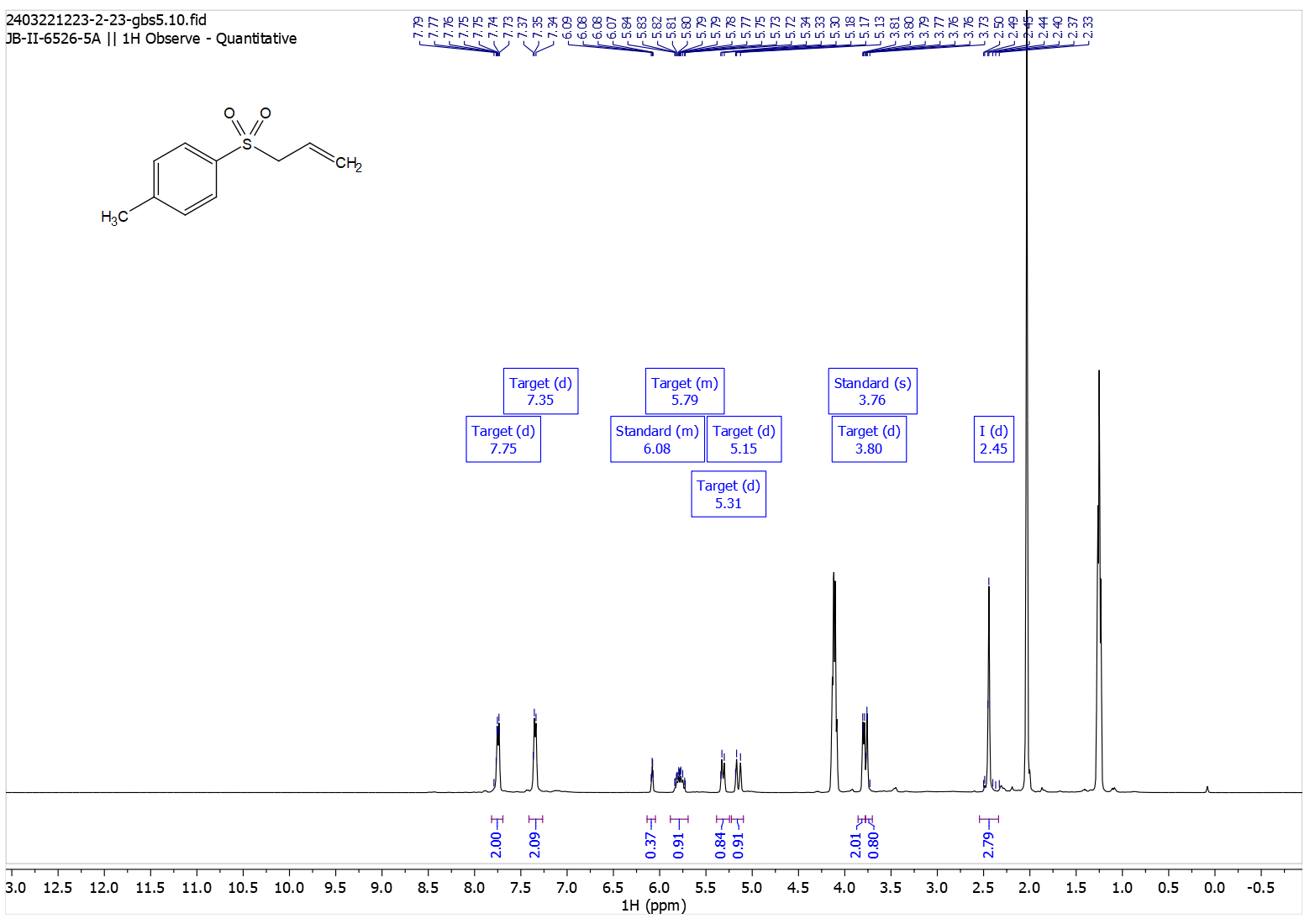
**

Figure **S13**. Example of ^1^H NMR spectrum of crude reaction mixture from Reaction 1b in CDCl_3_.

**
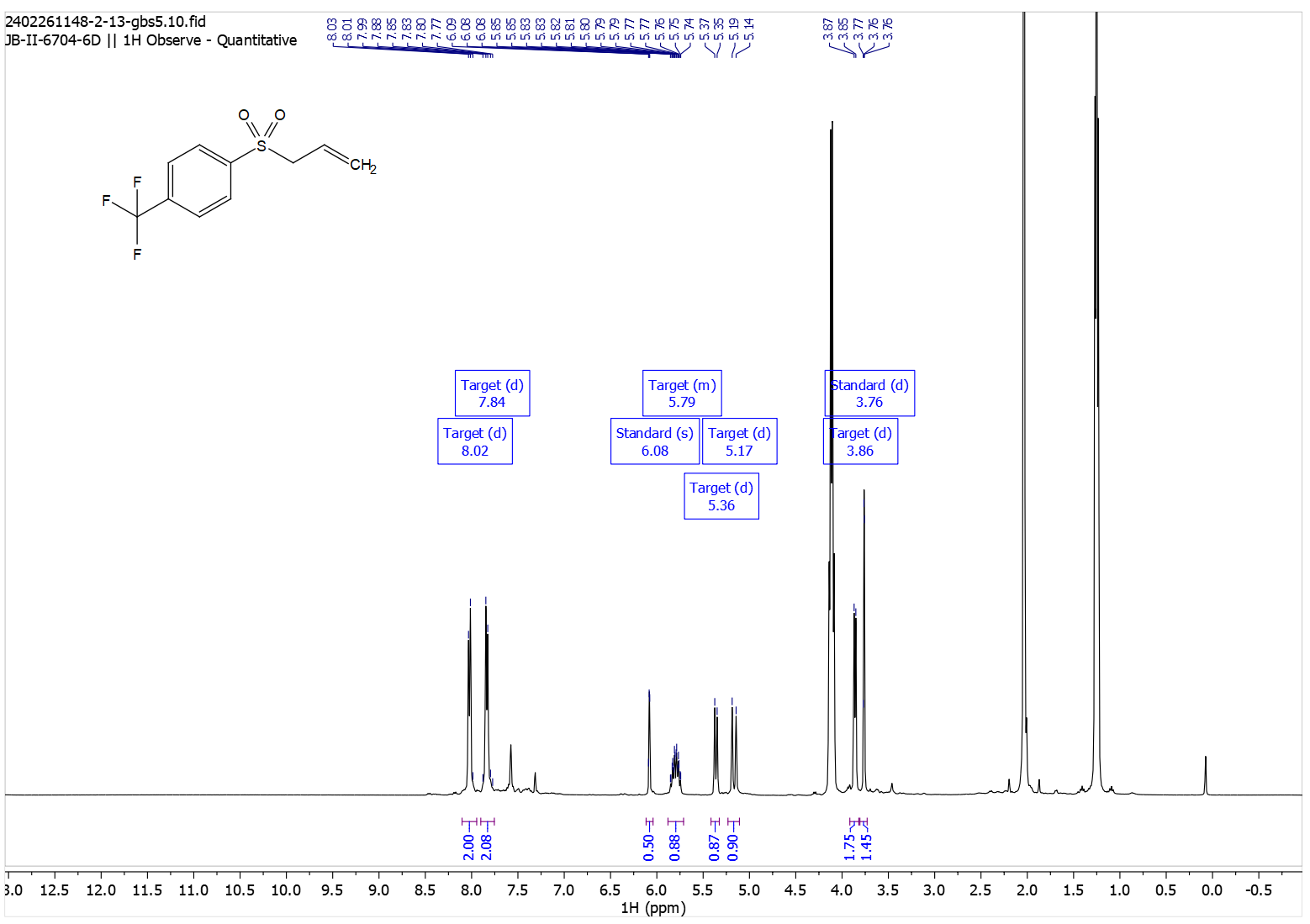
**

Figure **S14**. Example of ^1^H NMR spectrum of crude reaction mixture from Reaction 1c in CDCl_3_.

**
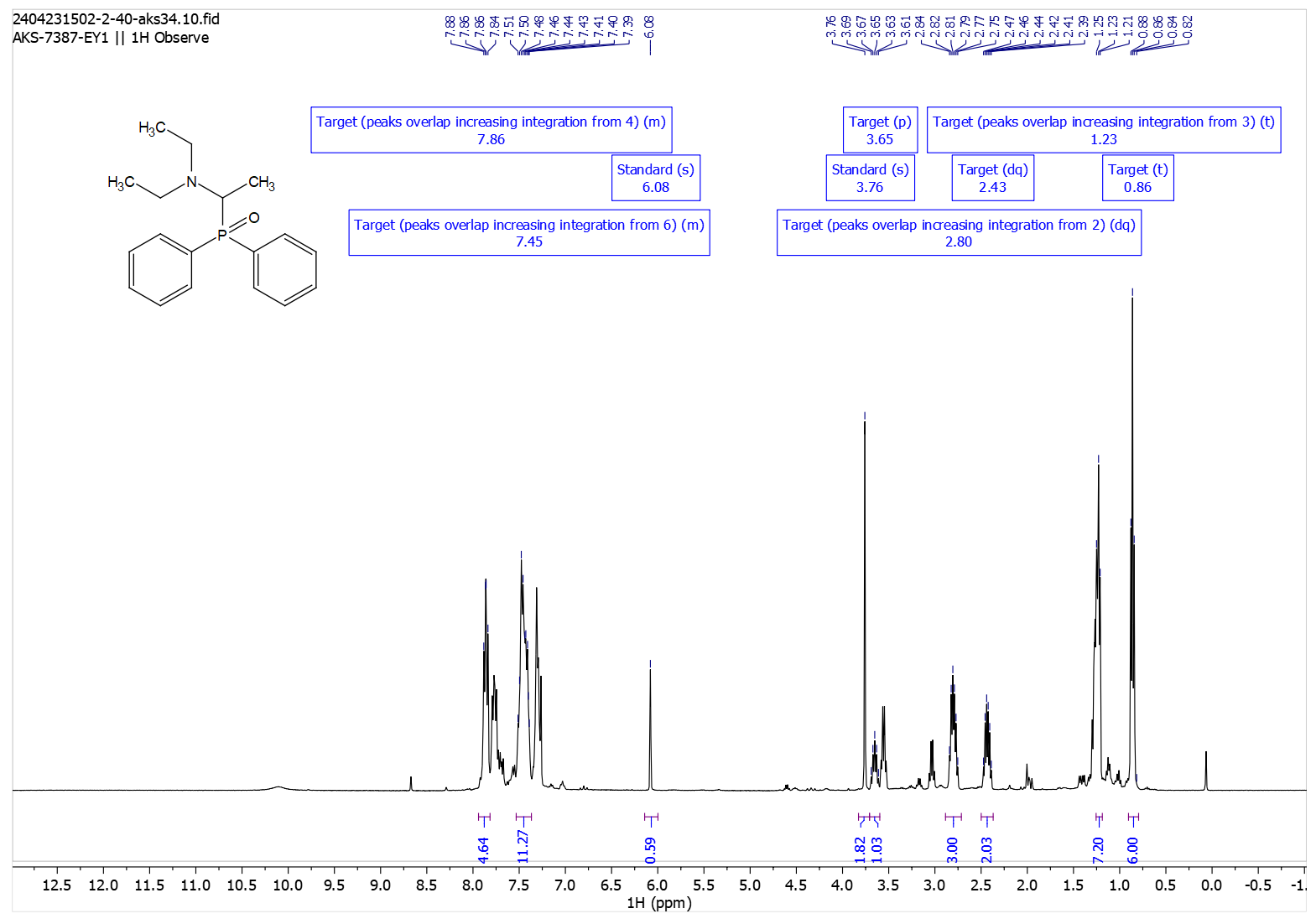
**

Figure **S15**. Example of ^1^H NMR spectrum of crude reaction mixture from Reaction 2 in CDCl_3_.

**
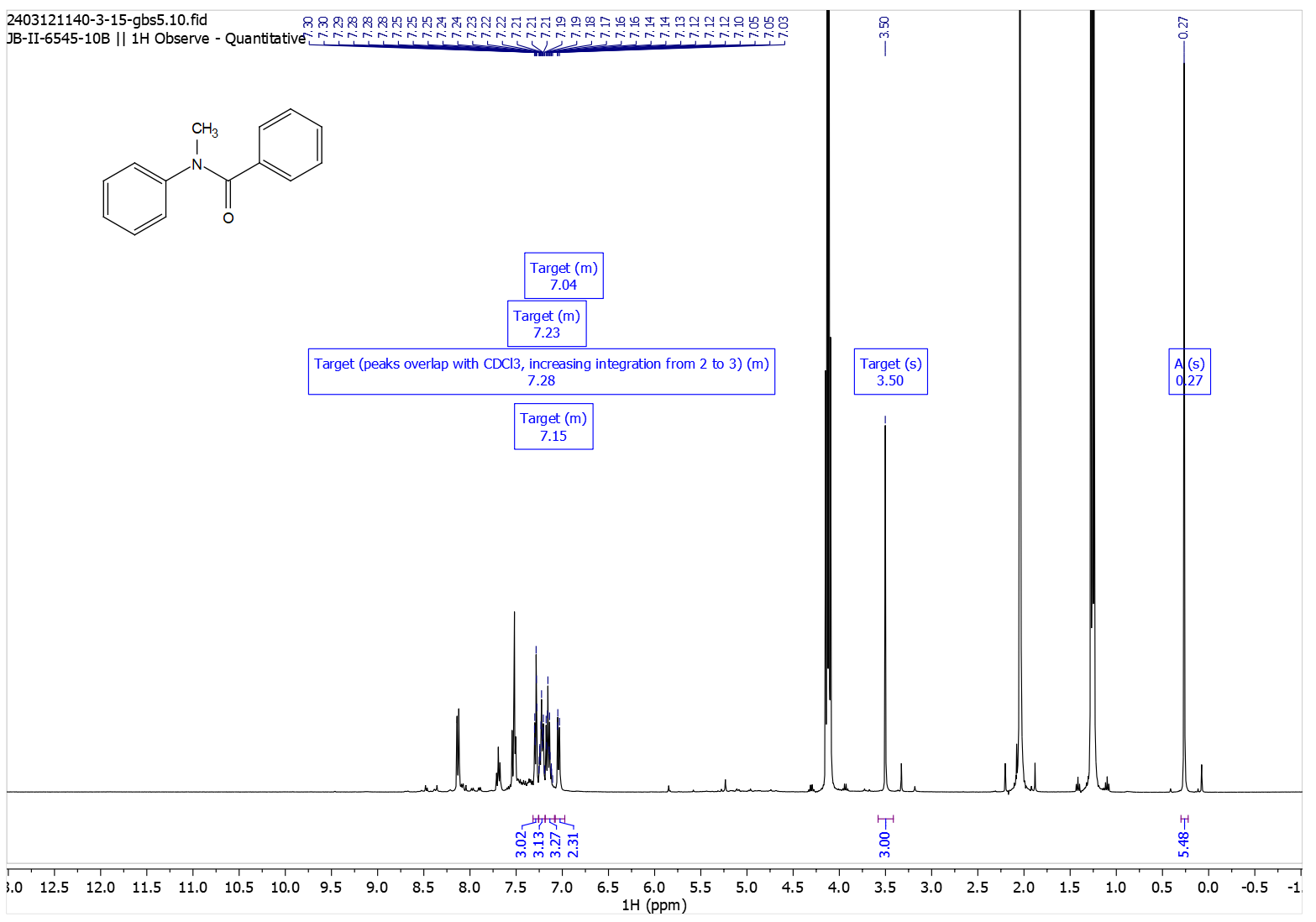
**

Figure **S16**. Example of ^1^H NMR spectrum of crude reaction mixture from Reaction 3 in CDCl_3_.

**
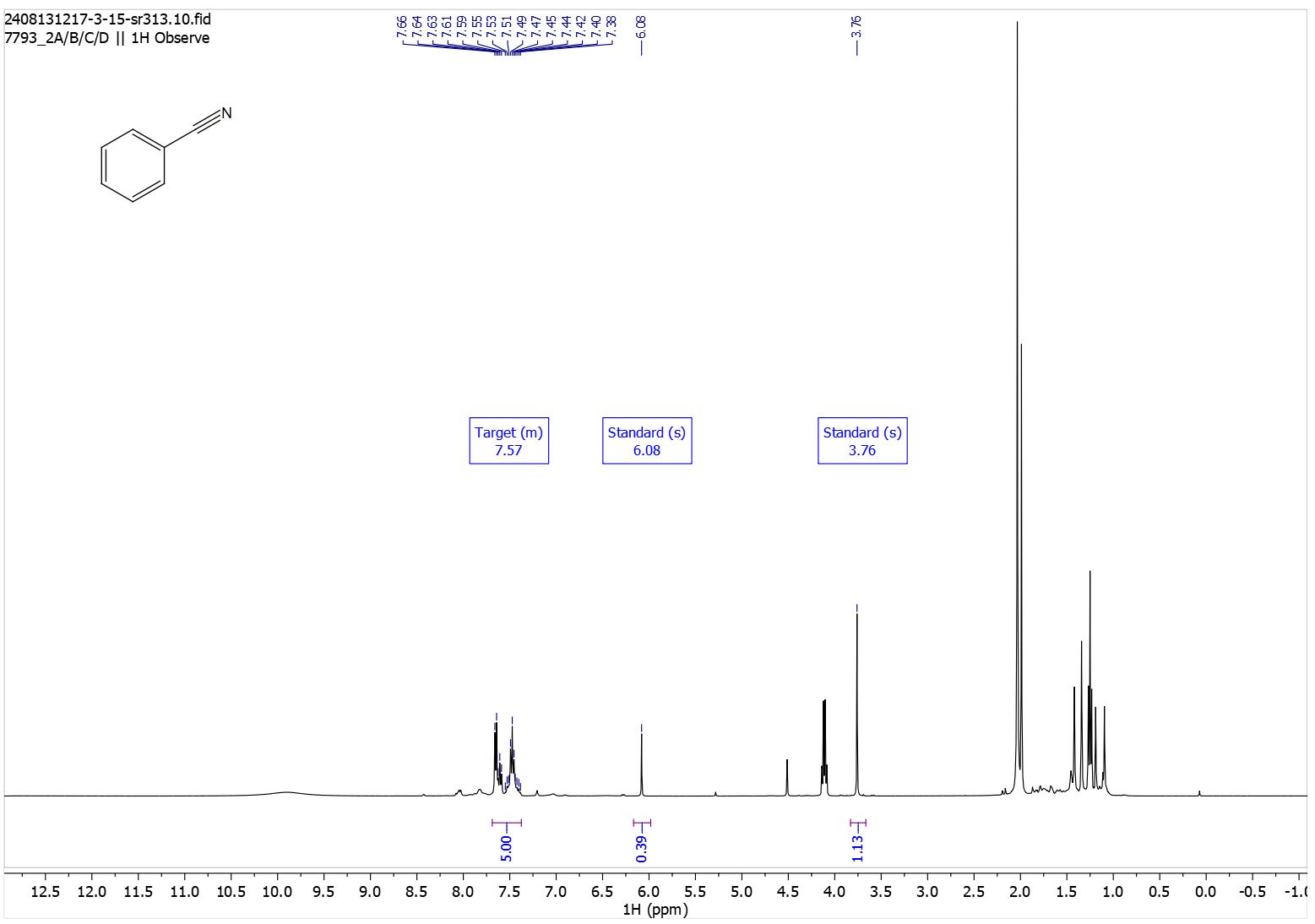
**

Figure **S17**. Example of ^1^H NMR spectrum of crude reaction mixture from Reaction 4 in CDCl_3_.

**
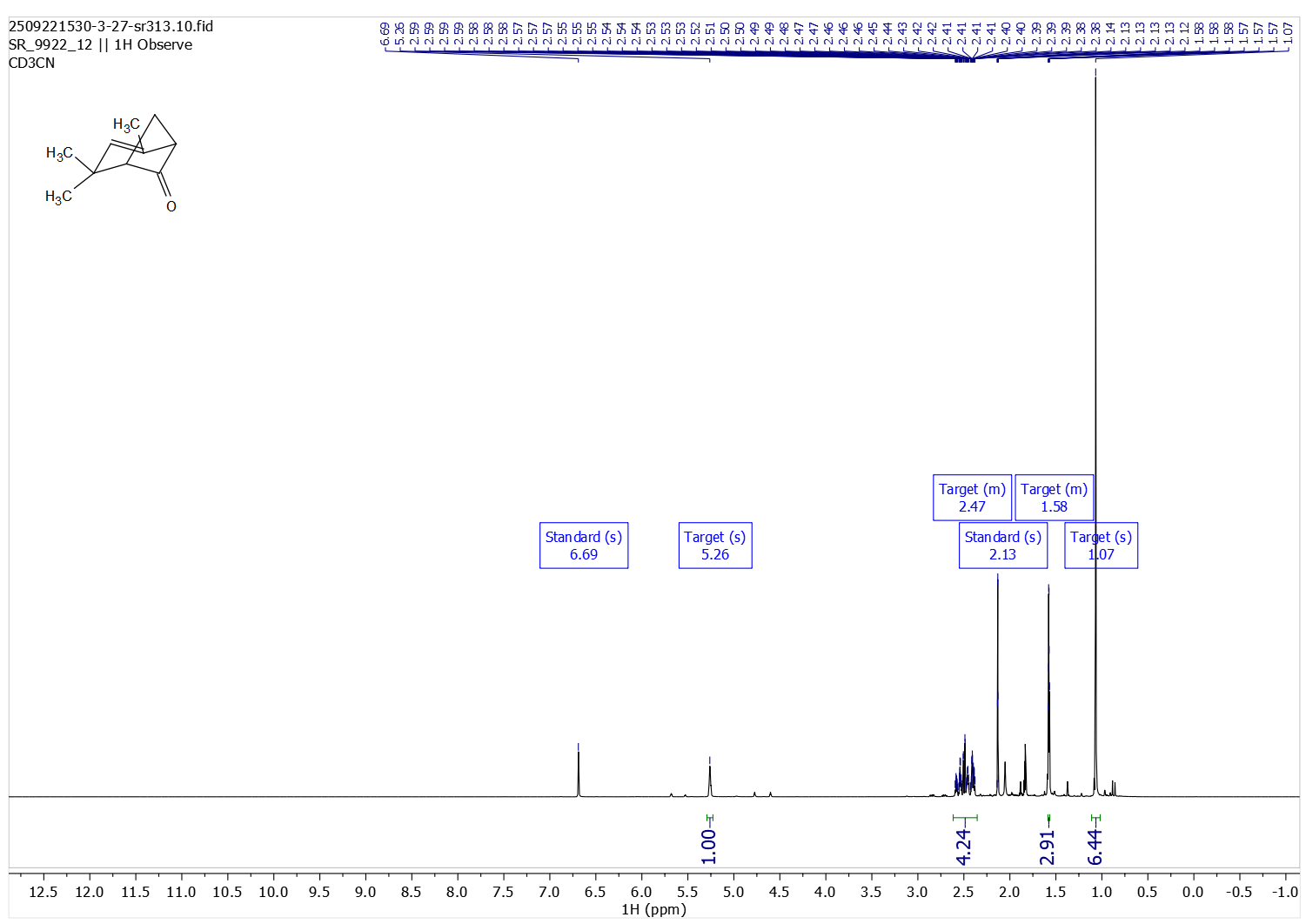
**

Figure **S18**. Example of ^1^H NMR spectrum of crude reaction mixture from Reaction 5 in MeCN-*d*_3_.

**References**

(1) Bu, M.-J.; Cai, C.; Gallou, F.; Lipshutz, B. H. PQS-enabled visible-light iridium photoredox catalysis in water at room temperature. *Green Chem.* **2018**, *20*, 1233-1237.

(2) Kim, S.; Rojas-Martin, J.; Toste, F. D. Visible light-mediated gold-catalysed carbon(sp2)–carbon(sp) cross-coupling. *Chem. Sci.* **2016**, *7*, 85-88.

(3) Hu, C.; Chen, Y. Chemoselective and fast decarboxylative allylation by photoredox catalysis under mild conditions. *Org. Chem. Front.* **2015**, *2*, 1352-1355.

(4) Vasu, D.; Fuentes de Arriba, A. L.; Leitch, J. A.; de Gombert, A.; Dixon, D. J. Primary α-tertiary amine synthesis via α-C–H functionalization. *Chem. Sci.* **2019**, *10*, 3401-3407.

(5) Speckmeier, E.; Fischer, T. G.; Zeitler, K. A Toolbox Approach To Construct Broadly Applicable Metal-Free Catalysts for Photoredox Chemistry: Deliberate Tuning of Redox Potentials and Importance of Halogens in Donor–Acceptor Cyanoarenes. *J. Am. Chem. Soc.* **2018**, *140*, 15353-15365.

(6) Rawner, T.; Lutsker, E.; Kaiser, C. A.; Reiser, O. The Different Faces of Photoredox Catalysts: Visible-Light-Mediated Atom Transfer Radical Addition (ATRA) Reactions of Perfluoroalkyl Iodides with Styrenes and Phenylacetylenes. *ACS Catal.* **2018**, *8*, 3950-3956.

(7) Wang, C.; Guo, M.; Qi, R.; Shang, Q.; Liu, Q.; Wang, S.; Zhao, L.; Wang, R.; Xu, Z. Visible-Light-Driven, Copper-Catalyzed Decarboxylative C(sp3)−H Alkylation of Glycine and Peptides. *Angew. Chem. Int. Ed.* **2018**, *57*, 15841-15846.

(8) Bryden, M. A.; Crovini, E.; Comerford, T.; Studer, A.; Zysman-Colman, E. Organic Donor–Acceptor Thermally Activated Delayed Fluorescence Photocatalysts in the Photoinduced Dehalogenation of Aryl Halides. *Angew. Chem. Int. Ed.* **2024**, *63*, e202405081.

(9) Bryden, M. A.; Millward, F.; Matulaitis, T.; Chen, D.; Villa, M.; Fermi, A.; Cetin, S.; Ceroni, P.; Zysman-Colman, E. Moving Beyond Cyanoarene Thermally Activated Delayed Fluorescence Compounds as Photocatalysts: An Assessment of the Performance of a Pyrimidyl Sulfone Photocatalyst in Comparison to 4CzIPN. *J. Org. Chem.* **2022**, *88*, 6364-6373.

(10) Buettner, C. S.; Stavagna, C.; Tilby, M. J.; Górski, B.; Douglas, J. J.; Yasukawa, N.; Leonori, D. Synthesis and Suzuki–Miyaura Cross-Coupling of Alkyl Amine-Boranes. A Boryl Radical-Enabled Strategy. *J. Am. Chem. Soc.* **2024**, *146*, 24042-24052.

(11) Hämmerling, L.; Zysman-Colman, E. Building a photocatalyst library of MR-TADF compounds with tunable excited-state redox potentials. *Chem Catal.* **2024**, *4*, 101061.

(12) Liu, S.-P.; He, Y.-H.; Guan, Z. Photoredox-Catalyzed Radical–Radical Cross-Coupling of Sulfonyl Chlorides with Trifluoroborate Salts. *J. Org. Chem.* **2023**, *88*, 11161-11172.

(13) Mahmood, Z.; He, J.; Cai, S.; Yuan, Z.; Liang, H.; Chen, Q.; Huo, Y.; König, B.; Ji, S. Tuning the Photocatalytic Performance of Ruthenium(II) Polypyridine Complexes Via Ligand Modification for Visible‐Light‐Induced Phosphorylation of Tertiary Aliphatic Amines. *Chem. Eur. J.* **2023**, *29*, e202202677.

(14) Liu, C.; Chen, H.-N.; Xiao, T.-F.; Hu, X.-Q.; Xu, P.-F.; Xu, G.-Q. Organic photoredox catalyzed dealkylation/acylation of tertiary amines to access amides. *Chem. Commun.* **2023**, *59*, 2003-2006.

(15) He, X.; Zheng, Y.-W.; Chen, B.; Feng, K.; Tung, C.-H.; Wu, L.-Z. Metal-free synthesis of nitriles from aldehydes and ammonium by visible-light photocatalysis. *Sci. China Chem.* **2023**, *66*, 2852-2857.

(16) Griffin, M.; Zysman-Colman, E. High Triplet Energy Iridium(III) NHC Complexes as Photocatalysts. *ChemRxiv.* **2025**, 10.26434/chemrxiv-22025-t26373h.

(17) Schmid, L.; Glaser, F.; Schaer, R.; Wenger, O. S. High Triplet Energy Iridium(III) Isocyanoborato Complex for Photochemical Upconversion, Photoredox and Energy Transfer Catalysis. *J. Am. Chem. Soc.* **2022**, *144*, 963-976.
